# HIV reprograms host m^6^Am RNA methylome by viral Vpr protein-mediated degradation of PCIF1

**DOI:** 10.1038/s41467-021-25683-4

**Published:** 2021-09-20

**Authors:** Qiong Zhang, Yuqi Kang, Shaobo Wang, Gwendolyn Michelle Gonzalez, Wanyu Li, Hui Hui, Yinsheng Wang, Tariq M. Rana

**Affiliations:** 1 Division of Genetics, Department of Pediatrics, Institute for Genomic Medicine, Program in Immunology, Center for AIDS Research, University of California San Diego School of Medicine, 9500 Gilman Drive, La Jolla, CA 92093 USA; 2 Department of Biology, Bioinformatics Program, University of California San Diego School of Medicine, 9500 Gilman Drive, La Jolla, CA 92093 USA; 3 Environmental Toxicology Graduate Program and Department of Chemistry, University of California, Riverside, CA 92521 USA

**Keywords:** HIV infections, RNA metabolism

## Abstract

*N*^6^,2′-*O*-dimethyladenosine (m^6^Am) is an abundant RNA modification located adjacent to the 5′-end of the mRNA 7-methylguanosine (m^7^G) cap structure. m^6^A methylation on 2′-*O*-methylated A at the 5′-ends of mRNAs is catalyzed by the methyltransferase Phosphorylated CTD Interacting Factor 1 (PCIF1). The role of m^6^Am and the function of PCIF1 in regulating host–pathogens interactions are unknown. Here, we investigate the dynamics and reprogramming of the host m^6^Am RNA methylome during HIV infection. We show that HIV infection induces a dramatic decrease in m^6^Am of cellular mRNAs. By using PCIF1 depleted T cells, we identify 2237 m^6^Am genes and 854 are affected by HIV infection. Strikingly, we find that PCIF1 methyltransferase function restricts HIV replication. Further mechanism studies show that HIV viral protein R (Vpr) interacts with PCIF1 and induces PCIF1 ubiquitination and degradation. Among the m^6^Am genes, we find that PCIF1 inhibits HIV infection by enhancing a transcription factor ETS1 (ETS Proto-Oncogene 1, transcription factor) stability that binds HIV promoter to regulate viral transcription. Altogether, our study discovers the role of PCIF1 in HIV–host interactions, identifies m^6^Am modified genes in T cells which are affected by viral infection, and reveals how HIV regulates host RNA epitranscriptomics through PCIF1 degradation.

## Abstract (web summary)

m6Am is a modification of the 5′ end of mRNAs catalyzed by PCIF1. Here, Zhang et al. show that HIV infection induces a decrease in m6Am of cellular mRNAs through Vpr-mediated PCIF1 ubiquitination and degradation, resulting in increased HIV replication through regulation of host transcription factors.

## Introduction

RNA contains more than 100 chemical modifications and recent studies on the structure and functions of these modifications have led to a new frontier in biology and medicine termed epitranscriptomics. One of these modifications, *N*^6^-methyladenosine (m^6^A) is the most prevalent RNA modification in many species, including mammals, and is found in 5′-UTR, 3′-UTRs, and stop codons^1–3^. The m^6^A modification is catalyzed by the RNA methyltransferase complex containing METTL3 that catalyzes the addition of a methyl group at the *N*^6^ position of adenosine which affects gene expression via regulation of RNA metabolism, function, and localization^4,5^. Another abundant RNA modification near the mRNA cap structure is dimethylated adenosine, *N*^6^,2′-*O*-dimethyladenosine (m^6^Am)^6,7^. Since m^6^Am is found at the first transcribed nucleotide in ~30% of the cellular mRNAs, m^6^Am can have a major influence on the gene expression of the transcriptome^7^. Recent studies have identified the Phosphorylated CTD Interacting Factor 1 (PCIF1) as the enzyme that catalyzes m^6^A methylation on 2′-*O*-methylated A at the 5′-ends of mRNAs^8–11^.

RNA modifications including m^6^A play important roles in modulating host–viral interactions^12–14^. Previous studies have found that diverse viral transcripts can be modified by m^6^A, thus affecting viral replication by a variety of mechanisms involving transcription, splicing, stability or export of viral RNA^15–23^. In addition, flavivirus infections such as Zika, West Nile, Dengue, and hepatitis C viruses alter the m^6^A in cellular mRNAs^24,25^, and m^6^A affects viral propagation by regulating host factors^24,25^. The SARS-CoV-2 virus also has m^6^A modifications, which are enriched in the 3′ end of the viral genome^26^. m^6^A reduction in viral RNA, by depleting host cell m^6^A methyltransferase METTL3, increased RIG-I binding and subsequently enhanced the downstream innate immune signaling pathway and inflammatory gene expression^26^. HIV genomic RNA can be modified by diverse RNA modifications, including Am, m^6^A, and m^5^C^19,21,23,27,28^. Recently, Tsai et al.^14^ reported that HIV RNA is acetylated to ac4C by cellular NAT10 and this modification enhances HIV gene expression through increased viral RNA stability.

So far, the role of m^6^Am RNA modification and the catalytic function of PCIF1 in biological and disease processes, especially in regulating viral infections and host–pathogens interactions have not to be determined. Here we report that PCIF1 restricts HIV replication and is degraded by viral protein Vpr, which leads to an impaired m^6^Am modification of cellular mRNA. PCIF1 inhibits HIV infection by enhancing a transcription factor ETS1 (ETS Proto-Oncogene 1, transcription factor) stability that binds to the HIV promoter to regulate viral transcription.

## Results

### m^6^Am modification of cellular mRNA is decreased by HIV infection and is mediated by Vpr-induced PCIF1 degradation

**Fig. 1:**
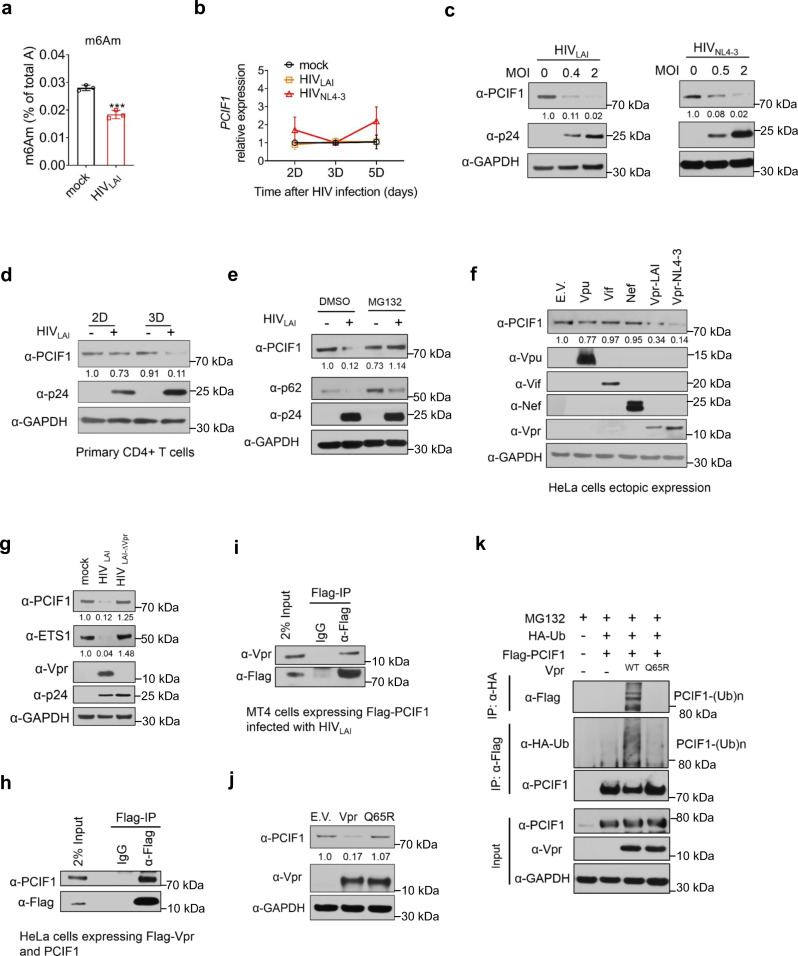
HIV infection downregulates m^6^Am modification of cellular mRNA by Vpr-induced degradation of the m^6^Am methyltransferase PCIF1. **a** m^6^Am modification of cellular mRNA is decreased by HIV infection. m^6^Am levels were quantified in MT4 cells infected with HIV_LAI_ (MOI = 0.4, 3 days) by LC–MS/MS. *n* = 3 biological independent experiments. Two-sided *t*-test. Mean ± SD, ***p = 0.0008. **b** *PCIF1* mRNA levels are not changed by HIV infection. *PCIF1* mRNA levels were quantified in MT4 cells infected with HIV_LAI_ (MOI = 0.4) or HIV_NL4-3_ (MOI = 2). *n* = 3 biological independent experiments. Two-sided *t*-test. Mean ± SD. **c** HIV infection decreases PCIF1 in MT4 cells. Immunoblotting of PCIF1 and p24 in MT4 cells infected with HIV_LAI_ or HIV_NL4-3_ for 3 days. **d** HIV infection decreases PCIF1 in primary CD4^+^ T cells. Immunoblotting of PCIF1 and p24 in activated primary CD4^+^ T cells infected with HIV_LAI_ (MOI = 1). **e** PCIF1 is downregulated by HIV through proteasome degradation. Immunoblotting of PCIF1, p62, and p24 in MT4 cells infected with HIV_LAI_ (MOI = 0.4, 3 days). Cells were incubated with DMSO or MG132 (0.25 µM) at 1 day before lysis. **f** PCIF1 is degraded by HIV viral protein Vpr. Immunoblotting of PCIF1 and the viral proteins in HeLa cells transfected with indicated expression vectors (E.V: empty vector). **g** Vpr deleted HIV does not degrade PCIF1 and ETS1 protein expression. Immunoblotting of indicated proteins in MT4 cells infected with HIV_LAI_ or HIV_LAI_ Vpr deleted virus (HIV_LAI-∆Vpr_) (MOI = 0.4, 3 days). **h** Vpr interacts with PCIF1. Flag immunoprecipitation was performed in HeLa cells co-transfected with plasmids expressing PCIF1 and Flag-tagged Vpr for 2 days. PCIF1 and Vpr expression and enrichment were detected by western blotting. **i** Vpr interacts with PCIF1 in T cells. MT4 cells expressing Flag-PCIF1 was infected with HIV (MOI = 1) for 3 days followed by Flag immunoprecipitation. Flag-PCIF1 and Vpr expression and enrichment were detected by western blotting. **j** The E3 complex binding site of Vpr is necessary to decrease PCIF1. Immunoblotting of PCIF1 and Vpr in HeLa cells transfected with plasmids expressing empty vector (E.V.), Vpr, or Vpr-Q65R mutant vector for 2 days. **k** Vpr induces PCIF1 ubiquitination. HA or FLAG immunoprecipitation was performed in HeLa cells co-transfected with plasmids expressing Flag-PCIF1, HA-Ub, either Vpr or Vpr-Q65R mutant for 2 days. HA-tagged ubiquitin, Flag-tagged PCIF1, and Vpr expression were detected by western blotting. Similar results were obtained from three independent experiments, and GAPDH expression was shown as a loading control (**c**–**j**).

To investigate the mRNA methylation by HIV infection, we infected MT4 T cells with HIV and quantified m^6^Am and m^6^A modifications in cellular mRNA by LC–MS/MS^26,29^. We found that m^6^Am modification of cellular mRNAs, but not m^6^A, was significantly decreased upon HIV-1 infection in T cells (Fig. 1a and Supplementary Fig. 1a). Since PCIF1 is the only known enzyme that catalyzes m^6^Am RNA methylation^8–10^, we analyzed the RNA and protein expressions of PCIF1 in MT4 cells infected with either the HIV-1 LAI or NL4-3 strain. Our results showed that despite robust infection, neither of the two strains reduced the mRNA levels of PCIF1 (Fig. 1b and Supplementary Fig. 1b, Supplementary Data 1). On the other hand, protein levels of PCIF1 were significantly decreased by both of the two HIV strains in a dose-dependent manner in MT4 T cells (Fig. 1c). PCIF1 degradation was also observed in primary CD4^+^ T cells on days 2 and 3 post-infection (Fig. 1d). Further, PCIF1 degradation is not cell line restricted because HIV infection also dose-dependently downregulated PCIF1 protein in HeLa cells infected with an HIV pseudovirus with only one cycle of replication, suggesting that the degradation was dependent on HIV replication (Supplementary Fig. 1c). Altogether, these results demonstrate that m^6^Am RNA methylation was reduced due to HIV-mediated degradation of the methyltransferase protein PCIF1.

To reveal which degradation pathway is responsible for PCIF1 downregulation, MT4 cells were infected with HIV_LAI_ and then treated with proteasome or lysosome inhibitor. PCIF1 degradation induced by HIV infection was significantly rescued by the proteasome inhibitor, MG132, but not by lysosome inhibition. p62 is an autophagy substrate and was used as a reporter of proteasome and lysosome activity (Fig. 1e and Supplementary Fig. 2a). These results suggested that PCIF1 was degraded by HIV through a proteasome pathway. The HIV genome expresses six accessory proteins including Tat, Rev, Vpu, Vif, Nef, and Vpr, four of which (Vpu, Vif, Nef, and Vpr) are known to target host restriction proteins’ degradation^30^. To identify which of these protein(s) is involved in PCIF1 degradation, we overexpressed each of the four proteins (Vpu, Vif, Nef, and Vpr) in HeLa cells and analyzed PCIF1 levels. We found that Vpr from both HIV_LAI_ and HIV_NL4-3_ decreased PCIF1 expression (Fig. 1f). Furthermore, Vpr-induced PCIF1 degradation in a dose-dependent manner (Supplementary Fig. 2b). To confirm whether Vpr is the factor responsible for PCIF1 degradation, cells were inoculated with either the wild-type or Vpr deleted (∆Vpr) HIV. The expressions of p24 were comparable in both wild-type and ∆Vpr viruses, while the Vpr protein band was abolished and PCIF1 was not decreased by ∆Vpr HIV (Fig. 1g). To rule out the role of Vpu, we performed the same experiment as for ∆Vpr by using wild-type and ∆Vpu HIV. Our results showed that ∆Vpu was not able to rescue PCIF1 degradation (Supplementary Fig. 2c). Together, these results indicate that PCIF is degraded by the proteasome and suggest that HIV Vpr is the key protein involved in PCIF1 degradation.

Vpr interacts with host factors and reprograms Cullin4-VprBP (a Vpr binding protein) E3 ligase complexes to ubiquitinate and degrade host proteins^31,32^. To investigate the underlying mechanism of Vpr-induced degradation of PCIF1, we first performed co-immunoprecipitation (Co-IP) to determine Vpr and PCIF1 interactions. We transfected HeLa cells with plasmids expressing PCIF1 and Flag-tagged Vpr followed by Co-IP and western blotting. Our results showed that Vpr has the ability to interact with PCIF1 (Fig. 1h). To determine Vpr–PCIF1 interactions in T cells, we infected MT4 cells expressing Flag-tagged PCIF1 with HIV_LAI_ for 3 days followed by Flag-IP and immunoblotting. Our results showed a clear interaction between PCIF1 and Vpr (Fig. 1i). In addition, we found that both Vpr and PCIF1 were mainly in the nucleus and the degradation of PCIF1 was obvious in the nucleus (Supplementary Fig. 2d). To investigate whether Vpr reprograms Cullin4-VprBP complexes to ubiquitinate and degrade PCIF1, a Vpr mutant Q65R, which cannot bind to VprBP^33^, was constructed to determine its effect on PCIF1. The Q65R mutant abolished the ability of Vpr to induce PCIF1 degradation (Fig. 1j). Furthermore, MG132 treatment accumulated polyubiquitylated PCIF1 in cells expressing Vpr, but not the Vpr-Q65R mutant (Fig. 1k). Altogether, these results demonstrate that HIV-1 Vpr promotes PCIF1 ubiquitination and degradation by the proteasome in a VprBP E3 complex.

### PCIF1 restricts HIV infection by repressing viral replication

**Fig. 2:**
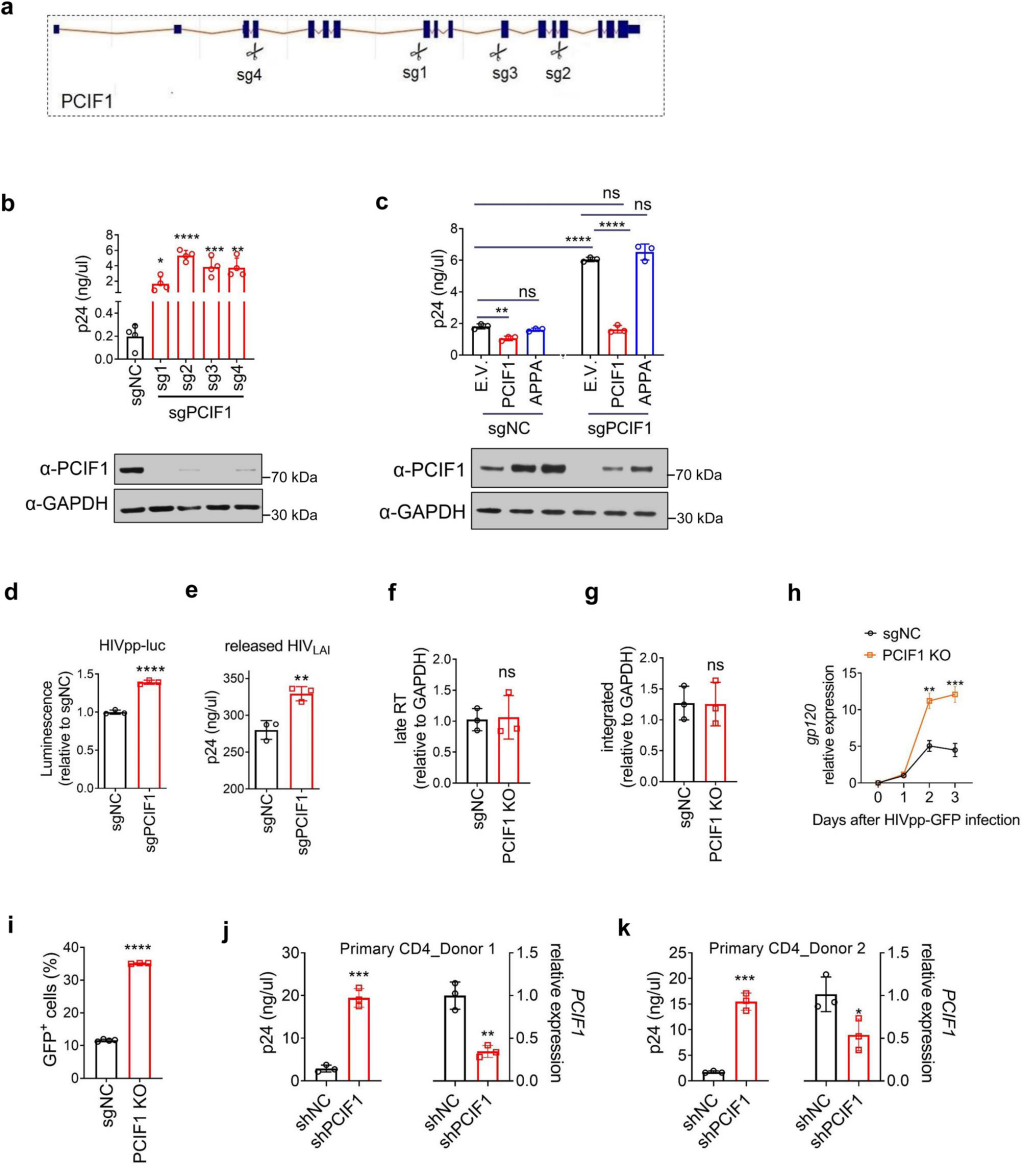
PCIF1 restricts HIV infection by inhibiting its transcription. **a**–**c** PCIF1 inhibits HIV. **a** Illustration of the 4 sgRNAs. **b**, **c** p24 ELISA was performed in MT4 cells transduced with indicated lentivirus vectors, and then infected with HIV_LAI_ (**b** MOI = 0.01, **c** MOI = 0.2) for 3 days. sgRNA resistant wild-type (PCIF1) or inactive mutant (APPA) was overexpressed in either control or PCIF1 KO cells. PCIF1 protein expressions were detected by western blotting. **b** *p = 0.02, ****p = 0.0000047, ***p = 0.0009, **p = 0.0013. **c** *p = 0.0036, ****p = 4.13E-06, ****p = 9.65701E-06, ns, not significant. **d**, **e** PCIF1 does not affect entry or release of HIV. The infection of HIV was detected using luciferase assays performed in PCIF1 knockout 293FT cells infected with HIV pseudovirus (HIVpp-luc, MOI = 0.2, 2 days). ****p = 0.000064. **e** p24 ELISA was performed in the supernatant of control or PCIF1 knockout 293FT cells transfected with HIV_LAI_ infectious clone (2 days). **p = 0.0061. **f**, **g** PCIF1 does not affect pre-integration and integration of HIV. Control and PCIF1 KO Jurkat cells were infected with single-cycle HIVpp-luc. The formation of late reverse transcriptase cDNA (late-RT) at 10 h (**f**) as well as integrated proviral DNA at 48 h (**g**) were assessed by qPCR. ns, not significant. **h**, **i** PCIF1 inhibits transcription but not translation of HIV. **h** Control or PCIF1 knockout Jurkat cells were infected with HIV pseudovirus (HIVpp-GFP, MOI = 0.2). HIV transcription was quantified by *gp120* RT-qPCR. **p = 0.0012, ***p = 0.00073. **i** HIV protein expression was detected by quantifying GFP reporter levels using flow cytometry (3 days). ****p = 1.4053E-08. **j**, **k** PCIF1 inhibits HIV replication in CD4^+^ primary T cell. Activated Primary CD4^+^ T cells from Donor 1 and 2 were transduced with PCIF1 shRNA or control shRNA (shNC). After re-activation, cells were infected with HIV_LAI_ for 3 days, p24 levels were measured by ELISA. *PCIF1* mRNA levels were detected by RT-qPCR. **j** ***p = 0.002, **p = 0.029. **k** ***p = 0.0002, *p = 0.04. All data are represented as mean ± SD and analyzed by a two-sided *t*-test in **b**–**k**. *n* = 3 (**c**–**k**), or 4 (**b**) independent experiments. Similar immunoblotting results were obtained from three independent experiments in **b** and **c**. GAPDH expression was shown as a loading control.

To investigate whether PCIF1 is involved in the HIV life cycle, we designed four sgRNAs to knockout (KO) PCIF1 in MT4 cells (Fig. 2a). sgRNA #1 shares the same sequences as reported by Boulias et al. ^9^. All four sgRNAs successfully abolished PCIF1’s expression (Fig. 2b). Compared to control cells, the expression of viral core protein p24 in PCIF1 KO cells was significantly increased (Fig. 2b). The RNA expressions of the HIV gp120 gene in both the infected cells and also in the released viral particles were also enhanced in KO cells (Supplementary Fig. 3a, b). These results suggest that PCIF1 restricts HIV infection. To further confirm these findings by another approach, we used two shRNAs constructs to knockdown PCIF1 expression. The PCIF1 mRNA and protein expressions were significantly interfered by shRNAs (Supplementary Fig. 3c). The released HIV particle, p24 protein expression, and gp120 RNA expressions were significantly enhanced by PCIF1 KD (Supplementary Fig. 3c, d).

PCIF1 consists of two core regions including methyltransferase and helicase domains. The active enzyme site with an NPPF motif in the methyltransferase is required for substrate recognition and catalysis^8^. To determine whether the methyltransferase activity of PCIF1 is responsible for HIV restriction, rescue experiments were performed by introducing either sgRNA resistant wild-type (WT) PCIF1 or catalytically inactive PCIF1 (APPA) back into the KO cells. In control cells, the overexpression of WT PCIF1, but not the APPA mutant, inhibited p24 expression. Moreover, in KO cells, while the expression of WT PCIF1 rescued HIV replication, the APPA mutant did not affect HIV replication (Fig. 2c and Supplementary Fig. 3e). These results confirm that catalytically active PCIF1 inhibits HIV infection.

**Fig. 3:**
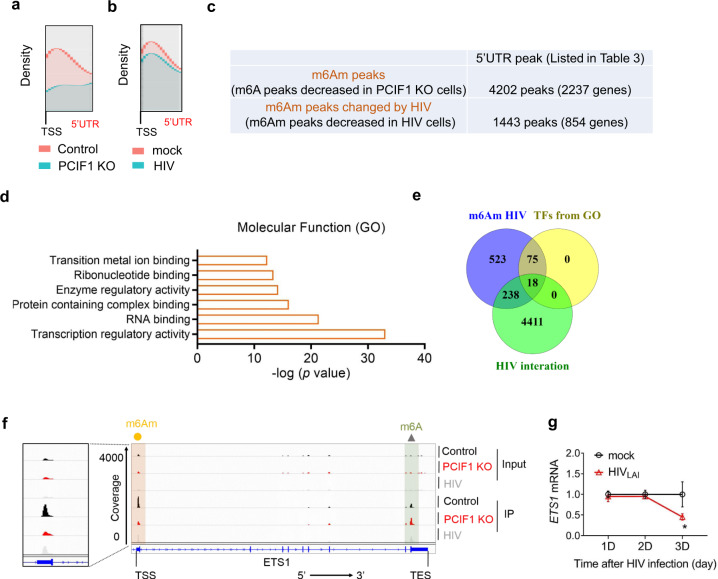
Identification of m^6^Am-modified cellular genes altered by HIV infection. **a** m^6^Am-Exo-MeRIP peaks in the 5′UTR are decreased in PCIF1 KO cells. Metagene analysis of m^6^Am-Exo-MeRIP peaks near the transcription start site (TSS) of all expressed genes in control or PCIF1 KO Jurkat cells. **b** m^6^Am-Exo-MeRIP peaks in the 5′UTR are reduced in HIV-infected cells. Metagene analysis of m^6^Am-Exo-MeRIP peaks in Jurkat cell mock-infected or infected with HIV_LAI_ at an MOI of 4. **c** m^6^Am peaks are altered by HIV infection. m^6^A-MeRIP peaks were aligned to the human genome. And peaks that are located in the 5′UTR and decreased in PCIF1 KO cells were considered as high-confidence m^6^Am peaks. Of them, peaks that are decreased by HIV infection were shown as potential m^6^Am peaks changed by HIV. All the peaks from **a**–**c** were conserved peaks called from two independent samples. **d** HIV-changed m^6^Am genes are enriched in the category of transcriptional factors. Molecular function GO analysis was performed in the 854 m^6^Am genes changed by HIV using GSEA (http://www.gsea-msigdb.org/gsea/msigdb/annotate.jsp). **e** Venny plot shown the 18 HIV altered m6Am targeted transcription factors. The 854 HIV-changed m6Am genes were overlapped the 93 transcription regulatory genes and the HIV interaction database (listed in Supplementary Table 4, https://www.ncbi.nlm.nih.gov/genome/viruses/retroviruses/hiv-1/interactions/). **f** m^6^Am-Exo-MeRIP peak of ETS1 gene in the 5′UTR is decreased in PCIF1 KO cells and HIV-infected cells. Genome tracks of the ETS1 gene were plotted with called m^6^A peaks. The m^6^Am peaks were enlarged in the right panel. One representative of two experiments is shown. **g** Kinetics of *ETS1* mRNA during HIV infection. *ETS1* mRNA expression was quantified in MT4 cells infected with HIV_LAI_ at MOI 0.4 for 1, 2, or 3 days. *n* = 3 independent experiments. Two-sided *t*-test. Mean ± SD. *p = 0.039.

To investigate which steps of the HIV life cycle are inhibited by PCIF1, we utilized two single-cycle viral infection systems and PCIF1 KO 293FT cells. In the first approach, we used HIV pseudovirus pre-packaged with VSV-G envelope protein, which lacks HIV envelope protein and thus cannot be packaged and released. Control (non-targeting sgRNA) or PCIF1 KO (sgPCIF1) 293FT cells were infected with pseudovirus (HIVpp-luc) and luciferase expression was quantified. Our results show that HIV infection was enhanced in PCIF1 KO cells (Fig. 2d and Supplementary Fig. 3f). Consistent with these findings, MT4 or Jurkat T cells, interfering PCIF1 expression using shRNAs also increased pseudovirus replications (Supplementary Fig. 3g, h). These results suggest that PCIF1 did not affect the packaging or release of HIV particles. To investigate whether PCIF1 acts on HIV entry into host cells, the infectious HIV_LAI_ cDNA vector was directly transfected into 293FT cells without the requirement of viral entry steps. The virus was replicated and released in control and KO cells. The released viral particles were quantified by p24 and a significant increase in the KO cells was observed, suggesting that PCIF1 did not affect the entry step of the HIV life cycle (Fig. 2e). After viral entry in cells, HIV genomic RNA is reverse-transcribed into viral DNA and subsequently integrated into the host genome by HIV integrase. To determine whether the pre-integration and integration were affected by PCIF1, a PCIF1 KO colony was selected, which was depleted of PCIF1 expression (Supplementary Fig. 3i). Control and PCIF1 KO Jurkat cells were infected with single-cycle HIVpp-luc. Total DNA was extracted at 10 and 48 h, and the formation of late reverse transcriptase cDNA (late-RT), as well as integrated proviral DNA, were assessed by qPCR (Fig. 2f, g). Comparative analysis of the formation of HIV late-RT cDNA at 10 h post-infection (Fig. 3f) showed little effect of PCIF1 on HIV pre-integration. Integrated proviral HIV DNA analysis through Alu-viral LTR qPCR showed that PCIF1 did not affect HIV integration at 48 h after infection (Fig. 3g). Thus, PCIF1 might affect the replication or translation phase of HIV. To analyze the replication and translation of HIV, the control and KO Jurkat T cells were infected with single-cycle pseudovirus HIVpp-GFP, and GFP expression was quantified. In addition, HIV RNA expression was analyzed during a single round of HIV replication. At day 2 post-infection, *gp120* RNA was significantly increased (~2-fold), and the effect was further enhanced to ~3-fold at 3-days post-infection (Fig. 2h). The GFP expression was correspondingly increased to 3-fold at 3-days post-infection (Fig. 2i). These results suggest that the replication of HIV was positively affected by PCIF1 depletion.

Next, we investigated PCIF1 function in primary CD4 T cells. Primary CD4 T cells were activated by using CD3 and CD28 antibodies. Two specific shRNAs targeting PCIF1 were mixed as a pool to increase the knockdown efficiency. Knockdown cells were enriched by puromycin selection for 5 days. Two donors were used to rule out individual differences. *PCIF1* mRNA levels were significantly reduced (Fig. 2j, k). Knockdown cells were then infected with HIV_LAI_ for 3 days. Consistent with results in MT4 and Jurkat T cells, knocking down PCIF1 increased p24 expression >7-fold in both donors (Fig. 2i). Next, we asked whether PCIF1’s ability to inhibit HIV is strain-type dependent. To address this question, we depleted PCIF1 in THP1 cells and differentiated them into macrophages followed by infection using HIV_BaL_, a CCR5-tropic strain. Our results show that the expression of gp120 mRNA was increased by PCIF1 depletion (Supplementary Fig. 3j, k). Taken together, these results show that PCIF1 is a broad HIV inhibitor that regulates CXCR4 and CXCR5-tropic viral replication in primary CD4 T cells and macrophages.

### PCIF1 does not methylate HIV genomic RNA

There are two possibilities for the mechanism through which PCIF1 regulates HIV transcription: (1) HIV transcripts are methylated by PCIF1 and thus affect their stability. (2) m^6^Am-modified host genes are changed by PCIF1 which then regulates the transcription of HIV. To investigate whether HIV transcripts are modified by PCIF1, we performed MeRIP-seq in control and PCIF1 KO Jurkat T cells infected with HIV. LC/MS–MS quantification of modified nucleotides in control and PCIF1 KO cells showed that m^6^Am was undetectable in the PCIF1 KO cells as compared to control cells, while m^6^A levels were unchanged in PCIF1 KO cells (Supplementary Fig. 4a, b). In vertebrates, the m^6^A modification is typically near the 3′ stop codons of mRNAs^2,3^, while m^6^Am modification is adjacent to the 5′ cap of mRNAs^6–10^. Three high-intensity m^6^A peaks were detected in the HIV genomic RNA. One was in the 5′UTR, the other two were near the 3′UTR. The peaks near the 3′UTR were predicted to be m^6^A peaks and were also detected in the previous studies^19,21,23^. Not surprisingly, the peaks near the 3′UTR were not changed by PCIF1 KO. Notably, the peak in the 5′UTR was also not changed in PCIF1 KO cells compared with control cells, suggesting that it was an m^6^A modification but not an m^6^Am modification (Supplementary Fig. 4c, d). These results suggest that PCIF1 does not modify HIV transcripts, and thus, rule out the first possibility for the mechanism through which PCIF1 regulates HIV transcription.

### Identification of m^6^Am-modified host genes

We then investigated the second possible mechanism for PCIF1’s regulation of HIV transcription. To determine which host genes are modified by PCIF1 and regulate HIV inhibition, we performed an m^6^A-methylated RNA immunoprecipitation sequencing (MeRIP-Seq) using m^6^A antibodies in control and PCIF1 KO Jurkat cell lines as well as cells infected with HIV (Supplementary Fig 5). As m^6^A and m^6^Am are structurally similar and both can be recognized by m^6^A antibodies, previous reports suggested that peaks in the 5′ end of the mRNAs are expected to contain m^6^Am modifications^34^. Consistent with previous observations^10^, the global m^6^A distribution in the gene body and 3′UTR were not drastically different from control cells (Supplementary Fig. 5a, b), suggesting that PCIF1 did not affect genome-wide m^6^A distribution. We observed a slight decrease in the 5′ end of mRNAs in the PCIF1 KO cells and HIV-infected cells (Supplementary Fig. 5a, b). We performed a motif analysis of the called peaks and all groups had DRACH (D = A, G, U; R A, G; H = A, C, U) m^6^A consensus as the common motif, suggesting that most of the peaks were m^6^A peaks (Supplementary Fig. 5c). These results suggest that the m^6^A MeRIP-Seq may not be an ideal method to directly identify m^6^Am-modified transcripts.

To identify transcriptome-wide m^6^Am enriched RNAs, we used m^6^Am-Exo-Seq, a recently developed methodology by Sendinc et al.^10^, in control and PCIF1 KO Jurkat cells. In parallel, m^6^Am-Exo-Seq in cells infected with HIV was performed to identify m^6^Am-modified genes that were altered by HIV infection. The density of m^6^A peaks was drastically decreased in the 5′UTR when PCIF1 was depleted (Fig. 3a). Additionally, in HIV-infected cells, we observed a significant decrease in the 5′UTR (Fig. 3b). To identify m^6^Am-modified genes in T cells, we compared the m^6^A peak in the 5′UTR in control and KO cells. The peaks that were depleted or decreased in the KO cells compared to control cells were considered to be m^6^Am peaks (Fig. 3c and Supplementary Data 2, 3). There were 4202 peaks in 2237 genes that were identified as m^6^Am peaks (Supplementary Data 2 and 3). A motif analysis of significant peaks showed the DRACH m^6^A consensus as to the most common motif in PCIF1 KO cells (Supplementary Fig. 6a). Previously, m6Am-Exo-Seq in the MEL624 melanoma cell line identified 1521 m^6^Am enriched genes^10^. Boulias et al., performed miCLIP-Seq in control and PCIF1 KO HEK293T cells and identified 1850 genes enriched in m^6^Am modification^9^. From our m^6^Am enriched genes in T cells, we found 425 and 539 genes common with m^6^Am-Exo-Seq and miCLIP-Seq results, respectively. There were 217 m^6^Am genes common in all three analyses, suggesting that these modifications may not be cell type-dependent. For example, PARP1, there was a significant peak near the annotated transcriptional starting site in the 5′UTR in control cells and was significantly depleted in KO cells (Supplementary Fig. 6b). Two of SMC1 isoforms with distinct TSS were both m^6^Am modified (Supplementary Fig. 6c). These data are consistent with the notion that the m^6^Am modifications are predominantly in TSS-proximal peaks.

### Identification of HIV-changed m^6^Am genes

Among 4202 m^6^Am genes, the m^6^Am peaks of 854 genes were decreased by HIV, while the other genes retained their peaks (Supplementary Data 3). These results further confirmed that HIV infection led to a large change in m^6^Am modification in T cells (Fig. 3c and Supplementary Fig 7, Supplementary Data 3, 4). Molecular functional analyses indicate that 93 of the 854 HIV-changed m^6^Am genes are in the category of transcription regulatory activity (Fig. 3d). As we confirmed that HIV transcription was inhibited by PCIF1 (Fig. 2d–i), we decided to focus on transcription factors for further mechanistic studies. To narrow down the most significant targets of PCIF1 that may affect HIV transcription, we overlapped the 93 transcription regulatory genes with an HIV interaction database (Supplementary Data 4, https://www.ncbi.nlm.nih.gov/genome/viruses/retroviruses/hiv-1/interactions/) and selected the targets with annotated interaction with HIV. There were 18 HIV-altered m^6^Am targeted transcription factors: ETS1, EGR1, JUND, SREBF2, PLSCR1, FOS, MYC, YY1, DDIT3, RARA, SP4, CEBPA, IKZF1, CDC5L, ATF4, MLLT1, RCOR1, and SMAD7, that were among the HIV interactors. For example, the peaks in the TSS of *ETS1* (Fig. 3f), *CDC5L* (Supplementary Fig. 7a), *DDIT3* (Supplementary Fig. 7b), *CEBPA* (Supplementary Fig. 7c), *FOS* (Supplementary Fig. 7d), and *MYC* (Supplementary Fig. 7e) were significantly abolished in HIV infected or PCIF1 KO group. Most of the genes are annotated to have TSS as adenosine except for *EGR1, YY1, DDIT3, CDC5L*, and *RCOR1*, which could be due to incorrect TSS annotation^9^, although we cannot exclude the possibility that they are not m^6^Am modified.

### PCIF1 increases ETS1 stability and restricts HIV replication

To investigate which genes are responsible for PCIF1-directed inhibition of HIV transcription, we first investigated whether the RNA levels of selected m^6^Am target genes were significantly changed by HIV. During HIV infection, the mRNA of *ETS1* was decreased at day 3 post-infection (Fig. 3g), which corresponds to a similar trend observed for PCIF1 degradation (Fig. 1), indicating that ETS1 was regulated by PCIF1 during HIV replication. The RNA levels of *FOS*, and *EGR1* were significantly increased as early as 1-day post-infection. Since PCIF1 was not significantly changed at day 1 post-infection, suggesting that PCIF1 may not be the only factor affecting *FOS* and *EGR1* expressions during HIV infection (Supplementary Fig. 8a). In addition, an unbiased high throughput yeast one-hybrid (eY1H) screen identified ETS1 interaction with HIV-1 LTR^35^. Therefore, we next investigated how ETS1 was regulated by PCIF1 and whether ETS1 can modulate HIV replication.

**Fig. 4:**
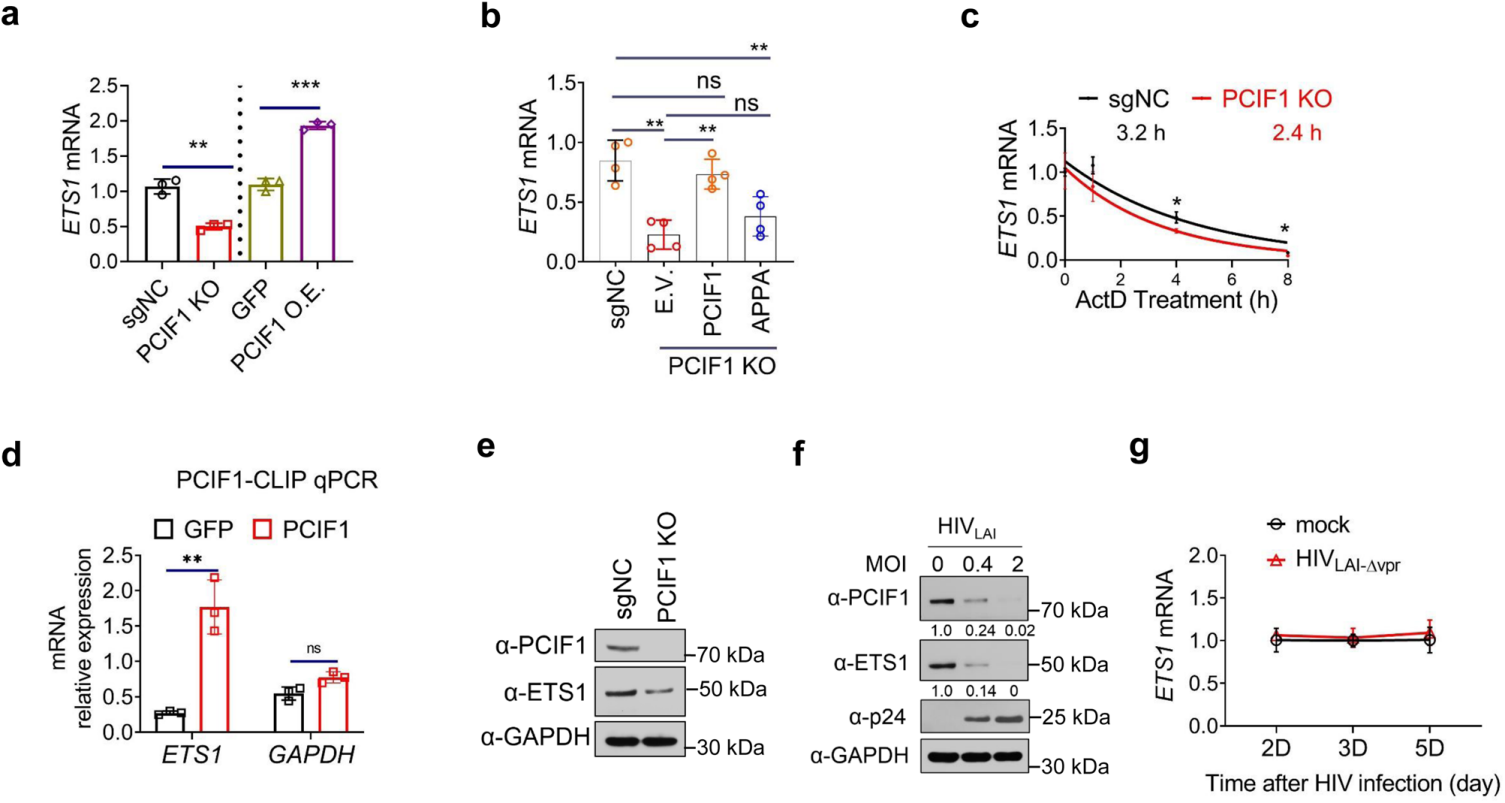
PCIF1 regulates ETS1 expression. **a** The mRNA expressions of *ETS1* was enhanced by PCIF1. The mRNA levels of the *ETS1* were analyzed using RT-qPCR in control and PCIF1 KO Jurkat cells, or PCIF1 overexpressed MT4 cells. **p = 0.001, ***p = 0.0001. **b** PCIF1 regulates ETS1 through its methyltransferase domain. *ETS1* mRNA was quantified by RT-qPCR in PCIF1 KO cells rescued with wild-type or methyltransferase inactivated PCIF1 mutant as in Fig. 2c. **p = 0.001, **p = 0.0012, **p = 0.0076, ns not significant. **c** ETS1 mRNA decay is accelerated in PCIF1 KO cells. Control or PCIF1 KO Jurkat cells were treated with Actinomycin D (ActD) for the indicated time points and cells were collected for RNA quantification. *p = 0.019, *p = 0.012. **d** CLIP-qPCR showing the association of *ETS1* transcript with PCIF1 in PCIF1 overexpressing MT4 cells. RNA and protein in control or Flag-PCIF1 MT4 cells were UV-crosslinked and sonicated. PCIF1 bound RNA was pulled down using Flag antibody. Bound protein and chromatin were enzyme digested and *ETS1* and *GAPDH* RNA were quantified by RT-qPCR. RNA levels were normalized to Input RNA. *p = 0.025. **e** Protein levels of ETS1 are decreased in PCIF1 KO cells. PCIF1 and ETS1 protein expression was detected in control and PCIF1 KO cells by western blotting. **f** ETS1 protein is decreased by HIV infection. PCIF1 and ETS1 protein expressions were analyzed in MT4 cells infected with HIV_LAI_ at an MOI of 0.4 or 2 for 3 days. **g** Kinetics of *ETS1* mRNA during HIV_LAI-∆Vpr_ infection. *ETS1* mRNA expression was quantified in MT4 cells infected with HIV_LAI-∆Vpr_ at MOI 0.4 for 2, 3, or 5 days. All data are represented as mean ± SD and analyzed by a two-sided *t*-test in **a**–**d**, **g**. *n* = 3 (**a**, **c**, **d**, **g**), or 4 (**b**) independent experiments. Similar results were obtained from three independent experiments in **e** and **f**.

In PCIF1 KO cells, mRNA expression of *ETS1* was decreased significantly compared to control cells, while in ectopic PCIF1 expressing T cells, mRNA expression of *ETS1* was increased ~2-fold compared to control cells (Fig. 4a). These data suggested that PCIF1 enhanced the mRNA expression of *ETS*1. To determine whether ETS1 is regulated by PCIF1 through its methyltransferase activity, wild-type PCIF1 or a catalytically inactive PCIF1 mutant was expressed in PCIF1 KO cells. Importantly, downregulation of *ETS1* mRNA in PCIF1 KO cells can be rescued by expressing wild-type PCIF1, but not catalytically inactive PCIF1 mutant, indicating that PCIF1 regulates *ETS1* expression and requires the methyltransferase activity of the enzyme (Fig. 4b).

RNA modifications could affect mRNA expression by changing its cytoplasmic export or stability. To investigate how m^6^Am regulates *ETS1* mRNA expression, the distribution and half-life of *ETS1* mRNA was quantified in control and PCIF1 KO cells. The localization of *ETS1* transcripts was not significantly changed in PCIF1 KO cells (Supplementary Fig. 9a). The half-life of *ETS1* mRNA in PCIF1 KO cells, however, was decreased from 3.2 to 2.4 h (Fig. 4c). Meanwhile, the expression and half-life of *GAPDH*, an un-modified gene, were not changed in PCIF1 KO cells (Supplementary Fig. 9b). In addition, crosslinking immunoprecipitation (CLIP) assays demonstrated the direct binding of PCIF1 to ETS1 transcripts (Fig. 4d). Altogether, these results suggest that ETS1 is m^6^Am modified by PCIF1 and the mRNA stability of *ETS1* is enhanced by m^6^Am modification. The regulation of ETS1 by PCIF1 is in agreement with the findings of Boulias et al. that m^6^Am modification enhanced the stability of a subset of mRNAs^9^.

Next, we determined the protein expression of ETS1 by immunoblotting. Consistent with the mRNA expression data, the protein levels of ETS1 were decreased in PCIF1 KO cells (Fig. 4e). Similar to the decrease seen in mRNA (Fig. 3g), protein expression of ETS1 was also downregulated in HIV-infected cells (Fig. 4f). Additionally, in Vpr deficient HIV-infected cells, *ETS1* mRNA was not changed (Fig. 4g and Supplementary Fig 9c), and its protein expression was rescued compared to wild-type HIV-infected cells (Fig. 1g). These data suggested PCIF1 and ETS1 are regulated by Vpr expressions. Altogether, our data strongly suggest that PCIF1 inhibits HIV infection through the methylation of *ETS1* mRNA.

**Fig. 5:**
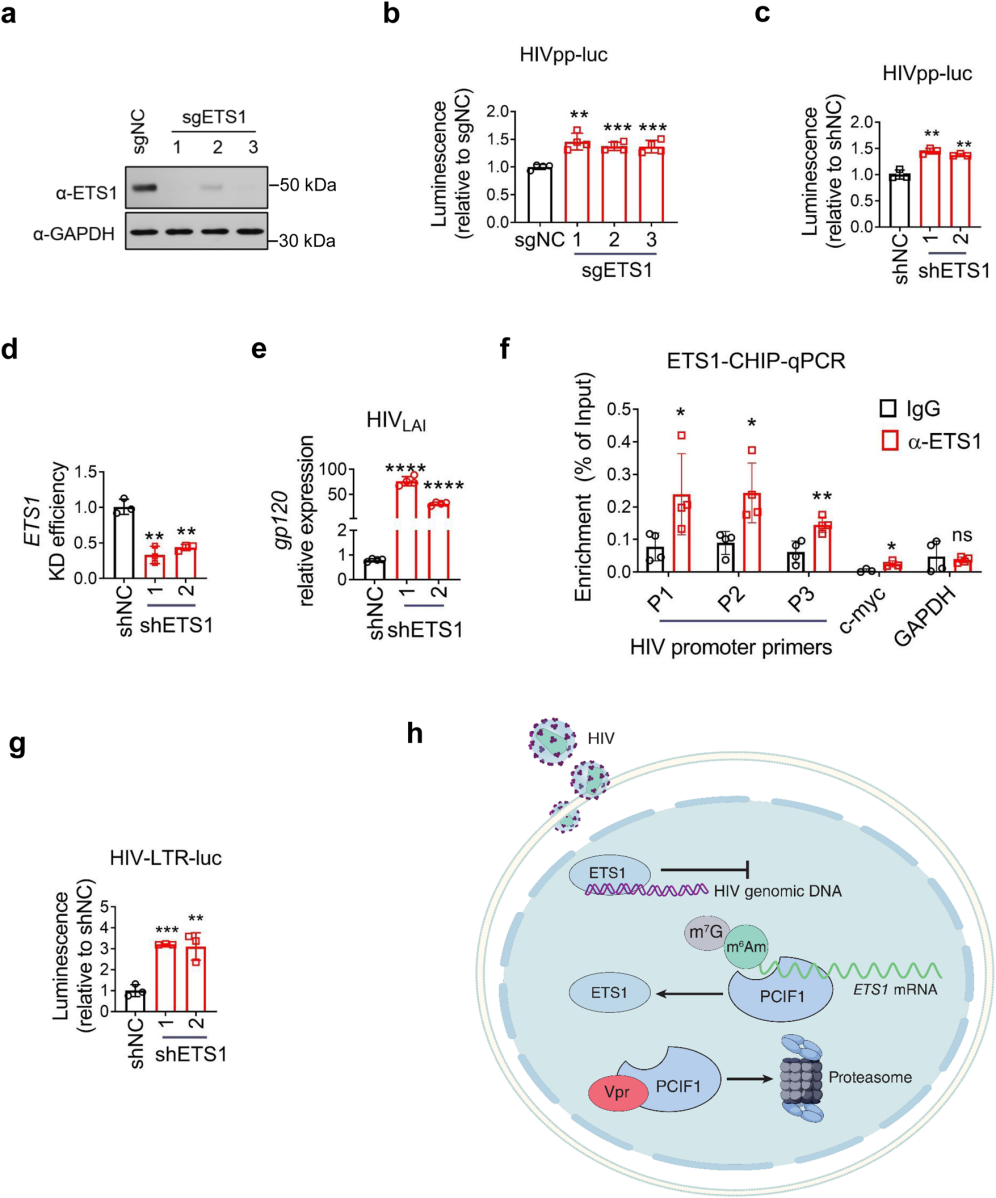
ETS1 inhibits HIV infection. **a**, **b** Knockout of ETS1 promotes HIV infection. MT4 were edited with ETS1 sgRNAs for 7 days, and then control or edited cells were infected with HIVpp-luc for 2 days. ETS1 protein levels were detected using western blotting (**g**). HIV replication was detected using luciferase assays (**h**). **p = 0.0012, ***p = 0.00015, ***p = 0.00094. **c**–**e** Interfering ETS1 expression increases HIV replication. Levels of single-cycle HIV infection were determined using luciferase assays in control shRNA or two ETS1 knockdown shRNA transduced MT4 cells infected with HIVpp-luc at an MOI of 0.2 for 2 days (**c**). *ETS1* (**d**) and *gp120* (**e**) mRNA expression levels were quantified in control shRNA or ETS1 KD MT4 cells infected with HIV_LAI_ at an MOI of 0.01 for 3 days. **c** **p = 0017, **p = 0021. **d** **p = 001, **p = 002. **e** ****p = 2.99781E−06, ****p = 1.20386E−06. **f** ETS1 is recruited to the HIV promoter. HIV-infected MT4 cells were prepared for ETS1-CHIP analysis. RT-qPCR of the HIV promoter regions or c-myc or GAPDH region coimmunoprecipitated with ETS was performed. *p = 0.049, *p = 0.020, **p = 0.007, *p = 0.0309, ns not significant. **g** ETS1 inhibits HIV promoter activity. A plasmid containing HIV 5′ LTR and a luciferase reporter (HIV-LTR-luc) was used to quantify HIV promoter activity. The reporter plasmid was electroplated into control or ETS1 KD MT4 cells and then protein expression was quantified using luciferase assay after 2 days. ***p = 0.00017, **p = 0.007. **h** Model illustrating Vpr–PCIF1-ETS1 regulation axis in HIV replication. During HIV replication, PCIF1 restricts HIV transcription and is targeted by viral protein Vpr for proteasome-mediated degradation, which leads to an impaired m^6^Am modification in cellular mRNAs. PCIF1 inhibits HIV infection by stabilizing a transcription factor ETS1 (ETS Proto-Oncogene 1, transcription factor) mRNA that binds HIV promoter to regulate viral transcription. All data are represented as mean ± SD and analyzed by a two-sided *t*-test in **b**–**g**. *n* = 3 (**c**, **d**, **g**), or 4 (**b**, **e**, **f**) independent experiments. Similar results were obtained from three independent experiments in **a**.

To elucidate the role of ETS1 in the HIV life cycle, we edited the *ETS1* gene using specific sgRNAs in MT4 cells, and then infected cells with HIV pseudovirus. ETS1 expression was successfully abolished in edited T cells (Fig. 5a) and the HIV infection was significantly increased in ETS1 edited cells (Fig. 5b), as observed in PCIF1 KO cells (Fig. 2d). Additionally, interfering ETS1 expression with two shRNAs significantly decreased ETS1 and increased HIV pseudovirus infection (Fig. 5c, d). The increase in HIV replication by ETS1 knockdown was enhanced when using multi-cycle HIV_LAI_ infection (Fig. 5e).

To confirm the binding of ETS1 to the HIV promoter, we analyzed ETS1 interactions with the HIV promoter by performing ETS1-CHIP experiments in HIV-infected MT4 cells. As shown in Fig. 4m, ETS1 exhibited significant interactions with the HIV promoter. ETS1 interactions with c-myc and GAPDH were used as positive and negative controls, respectively (Fig. 5f). To investigate the function of ETS1 on the HIV life cycle, we prepared an HIV reporter construct where the 5′LTR (long terminal repeat) containing regulatory modules was cloned in front of a luciferase gene and the promoter activity was monitored by luciferase expression. The HIV promoter-reporter (HIV-LTR-luc) was introduced into control or ETS1 knockdown cells and the luciferase activity was quantified as described above. We observed that the activity of the promoter was enhanced in ETS1 knockdown cells, suggesting that ETS1 reduced HIV transcription (Fig. 5g). All in all, these results demonstrate that ETS1 binds to the HIV promoter to decrease HIV transcription.

Finally, we asked if ETS1 expression contributes to HIV pathogenesis in people living with HIV (PLWH). We analyzed the mRNA expression of *ETS1* transcription factors in single-cell RNA sequencing data obtained from peripheral blood mononuclear cells from two healthy subjects (15,121 cells) and six HIV-infected donors (28,610 cells)^36^. Our analysis showed that ETS1 mRNA expression was reduced in the CD4 T cells of HIV-infected individuals as compared to healthy donors (Supplementary Fig. 10). Since ETS1 is high expressed in T cells, B cell, and NK cells and is necessary for T-cell survival and activation^37^, it is plausible that dysregulation of ETS1 in HIV-infected individuals causes an impairment in T-cell function. Taken together, our results show that PCIF1 restricts HIV infection through enhancing the stability of host m^6^Am genes including ETS1. HIV disarms this restriction by Vpr-induced degradation of PCIF1 during viral pathogenesis (Fig. 5h).

## Discussion

In this study, we demonstrate the role of m^6^Am methyltransferase, PCIF1, as an HIV restriction factor that is dependent on the methyltransferase activity of the enzyme. To counteract viral inhibition by PCIF1, HIV Vpr degrades PCIF1 proteins through a proteasome pathway. We identified 2237 m^6^Am genes in T cells, with most of their modifications abolished during HIV infection. We confirmed that PCIF1 enhanced the expression of ETS1, a transcriptional factor that was among the identified m^6^Am genes in T cells, which was decreased during HIV infection. We also found that ETS1 depletion promotes HIV transcription, suggesting that PCIF1 represses HIV replication by enhancing ETS1 stability.

HIV genomic RNA can be modified by diverse RNA modifications, including Am, m^6^A, and m^5^C^19,21,23,27,28^. Recently, Tsai et al.^38^ reported that HIV RNA is acetylated to ac4C by cellular NAT10 and that this modification enhances HIV gene expression by increasing viral RNA stability. m^6^A modification of HIV and its effects on viral replication have been previously reported^21,23,39^ with the most prominent m^6^A peaks are located in the 5′UTR and 3′UTR. m^6^A and m^6^Am are structurally similar and could not be distinguished by m6A-MeRIP-seq in previous studies. It is possible that m^6^A peaks in the 5′UTR also contain m^6^Am. To address this, we performed MeRIP-seq in control and PCIF1 KO cells infected with HIV. We observed three similar m^6^A peaks in both control and KO cells, indicating that the peaks represent m^6^A modification (Supplementary Fig. 4a–d). In addition, HIV genomic RNA lacks the typical cap-A structure for m^6^Am. Therefore, we conclude that HIV genomic RNA does not contain any m^6^Am modifications.

Epitranscriptomic changes to host transcripts during viral infection suggest that cellular mRNA modifications play important roles in host–pathogen interactions^15–23^. We observed that after HIV infection, m^6^Am modifications of many cellular mRNAs were decreased (Fig. 1a). Among the 2237 m^6^Am genes, the m^6^Am peaks of 854 genes were decreased by HIV, indicating that the m^6^Am modification in T cells was drastically affected by HIV infection (Fig. 3c). By focusing on genes in the DNA-binding transcription factor category of molecular functions, we identified ETS1, which we found to be regulated by PCIF1 enzymatic activity and to play an important role in HIV replication and potentially in immune cell physiology during infection. ETS1 belongs to the large ETS family of transcription factors (TFs); the family members have highly similar DNA-binding domains (DBDs) with the core binding site consisting of the GGAA/T motif. Each member of the ETS family can bind many target genes with varying selectivity in vivo. Although we show that ETS1 directly binds to the HIV promoter and regulates viral transcription (Fig. 5f, g), our study cannot rule out the possibility of indirect effects of ETS1 on cellular genes which could affect host–virus interactions. ETS1 is preferentially expressed in T and B cells and is necessary for T-cell activation and survival^37,40^. Based on the single-cell RNA seq data analysis related to HIV infection and immune cell exhaustion (Supplementary Fig. 10), we could infer that the decrease of ETS1 in HIV-infected individuals induces an impairment in CD4 T-cell function and contributes to HIV pathogenesis. We predict that by focusing on other molecular functions and genes affected by PCIF1 (Fig. 3), new proteins that regulate host–viral interactions will be discovered.

Vpr overcomes restriction to virus replication in non-cycling myeloid cell populations because HIV-1 replication is reduced in the absence of Vpr in myeloid cells such as monocyte-derived dendritic cells (MDDCs) and macrophages^41,42^. Several previous studies have reported the role of Vpr in modulating HIV replication in T cells^43–46^. Vpr drives systems-level proteomic remodeling by directly or indirectly targeting multiple proteins for degradation^47^. Recently, Bauby et al. provided a new perspective on Vpr function in primary CD4^+^ T cells by showing that Vpr-induced widespread transcription changes during early infection stages in T cells^48^. Furthermore, two reports demonstrate the functional significance of Vpr in HIV infection of T cells: (1) Vpx and Vpr overcome transcriptional repression of proviruses by the HUSH complex in Jurkat T cells^49^; (2) Vpr inhibits exonuclease 1-mediated restriction of HIV-1 Infection in T cells^50^. In our experiments using MT4 cells, we observed that replication of HIV _LAI-∆Vpr_ was increased to only 2-fold in 24 h, which is significantly lower than the 5-fold increase seen in the WT virus under the same time points of infection (Supplementary Figs. 1b and 9c). Altogether, these results suggest that Vpr promotes HIV replication in T cells, but its effects could be more moderate than those observed in myeloid cells. Since PCIF1 expression was comparable in macrophages and T cells, PCIF1 inhibits HIV in both THP1 derived macrophages, MT4 T cells, Jurkat T cells, and importantly primary CD4 T cells (Fig. 2 and Supplementary Fig. 3). Thus, PCIF1 is a broad inhibitor of HIV replication in macrophages and T cells that is counteracted by Vpr.

## Methods

### Cell culture and virus preparation

The procedures were approved by the Institutional Review Board. MT4, THP1, and Jurkat cells were cultured in RPMI containing 10% fetal bovine serum (FBS) and 50 µM β-mercaptoethanol (Sigma). HeLa and 293FT cells were cultured in DMEM (Invitrogen) with 10% FBS. To produce various viruses, HIV_LAI_ (pLAI.2, CAT# 2532, NIH AIDS Reagent Program), HIV_LAI-∆Vpr_ (A kind gift from Mario Stevenson), HIV_NL4-3_ (pNL4-3, CAT# 114, NIH AIDS Reagent Program), HIV_NL4-3-∆Vpu_ (pNL-U35, CAT# 968, NIH AIDS Reagent Program), HIV_pp-luc_ (pNL4-3.Luc.R-E-, CAT# 3418, NIH AIDS Reagent Program), and HIV_pp-GFP_ (CAT# 11100, NIH AIDS Reagent Program) virus, 293FT cells were seeded in a 10-cm plate at 6 × 10^5^ cells/ml. The viral vectors (15 μg) were transfected into 293FT cells using Lipofectamine 3000 (Invitrogen) and supernatants were collected after 2 days and centrifuged at 2000 g for 10 min to and filtered through 0.22 µm filters to remove debris. DNase I (100 U/ml, NEB) and RNase A (100 U/ml, Qiagen) were added and digested the free DNA and RNA in the supernatant at 37 °C for 1 h. Ultra-centrifuge (14,000×*g*, 4 h) was applied to enrich the viral particles. TRIzol was added into the precipitation and viral genomic RNA was extracted for LC–MS/MS analysis for RNA modifications.

### Primary CD4^+^ T-cell isolation and activation

Human peripheral blood mononuclear cells (PBMCs) were isolated from blood samples (purchased from San Diego Blood Bank) using Ficoll density centrifugation (Invitrogen) according to the manufacturer’s recommendations. The PBMC-containing interface layer was collected and washed with phosphate-buffered saline (PBS) three times. Then, primary CD4^+^ T cells were isolated using the EasySep Human CD4^+^ T-cell Enrichment kit (StemCell) following the manufacturer’s instructions. Isolated cells were cultured in a complete RPMI-1640 medium, which consisted of RPMI-1640 supplemented with 10% FBS, 20 U/ml IL-2, and 1% penicillin–streptomycin. To activate CD4^+^ T cells, 10 µl CD3 and CD28 T activator (StemCell) was added to 1 million cells for 1 week. To ensure robust infection, cells were re-activated with 10 µl activator per million cells for 24 h and then infected with HIV. To knockdown PCIF1 in primary T cells, activated Primary CD4^+^ T cells from Donor 1 and 2 were transduced with PCIF1 shRNA (shPCIF1) or control shRNA (shNC) and selected with puromycin (1.5 μg/ml) for 5 days.

### CRISPR mediated knockout

The 4 guide RNAs targeting PCIF1 are depicted in Fig. 2a. Knockout and characterization were performed according to previously described methods^51,52^. To knockout PCIF1 and ETS1, guide sequences (Supplementary Data 1) were synthesized and annealed for cloning into lentiCRISPR v2 (Addgene 52961). Positive clones containing guide sequences were sequenced. And 1.8 μg of guide RNA vectors along with psPAX (1.2 μg) and pMD2G (0.6 μg) were transfected into 1.2 million 293FT cells to produce lentivirus. MT4, Jurkat, or 293FT cells were transduced with lentivirus, puromycin (1.0 μg/ml) was added 24 h later to enrich the transduced cells, and the cells were cultured for 7 days to get edited. To pick the KO colonies in Jurkat and 293FT cells, the surviving cells were sorted into single-cell colonies in 96-well plates and cultured for 2 weeks. Positive KO clones were then detected with PCIF1 antibody using western blotting blot to confirm totally depletion of PCIF1.

### Vectors construction and HIV accessory protein expression

PCIF1 was cloned into plvx vector using in-fusion kit (Takara). PCIF1 knockout resistant vector and APPA mutated PCIF1 vector were generated using Q5 mutation kit (NEB). Vpr sequences from pLAI.2 vector were cloned into pFLAG-CMV plasmid. Q65 mutant was generated using Q5 point mutation kit. Primer sequences were listed in Supplementary Data 1. To express HIV accessory proteins in HeLa cells, the following vectors were obtained from the NIH AIDS reagent program: (1) HIV-1 NL4-3 Vif (pcDNA-HVif, Cat #10077), (2) HIV-1 NL4-3 Vpu and (pcDNA-Vphu, Cat #10076), (3) HIV-1 BRU/LAV GFP-Vpr (Cat #12478), (4) HIV-1 SF2 Nef (pcDNA3.1 SF2 Nef, Cat #11431), and (5) HIV-1 NL4-3 pEGFP-Vpr (Cat #11386). 1 μg of each vector was transfected into 0.3 million HeLa cells using lipofectamine 3000 for 2 days. And expressions of PCIF1 and accessory proteins were detected by western blotting blot. HA-Ub vector was purchased from Addgene (#18712). HIV_LAI_ LTR (−454 to +181) was cloned and inserted into pGL4.12 reporter vector. Vector was electroporated into MT4 cells and luciferase assay was performed two days later.

### mRNA purification and LC–MS/MS analysis for RNA modification

For the detection and quantification of m^6^A and m^6^Am modification of host mRNA, MT4 cells were infected with LAI at an MOI of 0.4 for 3 days. Total RNAs were extracted using TRIzol. Approximately 20 ug total RNA was applied to Poly(A) mRNA purification using mRNA isolation kit (NEB) according to the instruction, and the purification procedure was performed twice. To process the RNA for LC–MS/MS, 1.0 μg mRNA was de-capped with 1U Cap-Clip enzyme (Cellscript) at 37 °C for an hour. RNA modification levels were quantified using LC–MS/MS. First, 50 ng mRNA was digested by 0.5 unit of nuclease P1 (NP1) for 2 h at 37 °C. Followed by the addition of 0.25 unit alkaline phosphatase (CIP) and 3 μl of 1 M NH_4_HCO_3_ Buffer and incubated at 37 °C for 2 h. The digestion mixture was dried by speed vacuuming and reconstituted in 100 μl of ddH_2_O. For profiling and quantification of modified ribonucleoside by tandem mass spectrometry, 10 ng of digested RNA was added to labeled standards: 3.3 pmol of ^13^C_5_-labeled adenosine, 42.5 fmol of D_3_-N^6^-methyladenosine. All enzymes from the digestion mixture were removed by chloroform extraction. The aqueous layer was dried and reconstituted in 10 μl of ddH_2_O and 90 μl of acetonitrile to remove precipitates in that may block the nano-flow columns. This mixture was then incubated at −20 °C for 20 min, followed by drying by speed vacuuming to 90 μl. The samples were separated on a reverse-phase ultra-performance liquid chromatography C18 column with in-line mass spectrometry detection (Agilent 6410 QQQ triple-quadrupole LC mass spectrometer in positive electrospray ionization mode). For calibration curve construction, we calibrated using isotope-labeled standards that eluted at a similar time as nucleoside of interest. rA, m^6^A, and m^6^Am calibration curves employed in this analysis are presented in Supplementary Methods (Supplementary Tables 1 and 2; Supplementary Figs. 11 and 12).

### m^6^A-MeRIP-seq

Ten million control or PCIF1 KO Jurkat cells were mock-treated or infected with HIV LAI strain at an MOI of 4 for 3 days. Total RNA was extracted using TRIzol. About 200 μg RNA were then used to isolate poly(A) RNA using two rounds of oligo_25_ Dynabeads capture. For one sample, 10 μg poly(A) RNA was de-capped with 10U Cap-Clip enzyme and was used for m^6^A-MeRIP experiment. Briefly, RNA was fragmented and incubated with 5 μg of anti-m^6^A antibody (Abcam, ab151230) at 4 °C for 2 h. 100 μl protein G magnetic beads were added to pull down the m^6^A immunoprecipitation complex. After wash for three times, the bound RNA was eluted in 100 μl elution buffer and purified using RNA clean and concentrator-5 kit (Zymo Research). Final libraries were amplified and were subjected to paired-end sequencing in the HT sequencing core facilities.

### m^6^Am-Exo-Seq

In order to effectively map m^6^Am enrichment, we used a technique called m^6^Am-Exo-Seq developed by the Shi group^10^ with minor modifications. m6Am-Exo-Seq was performed in control or PCIF1 KO Jurkat cells, as well as in Jurkat cells infected with HIV at an MOI = 4. For one sample, 100 μg poly(A) RNA was fragmented in fragmenting buffer at 70 °C for 15 min. Fragmentation is stopped by the addition of EDTA to a final concentration of 70 mM. mRNA is purified with the RNA Clean and Concentrator-5 kit (Zymo Research). Uncapped and fragmented mRNAs are then phosphorylated by treating with 200 U of T4 PNK (NEB) in T4 ligase buffer for 90 min at 37 °C. Then, 20 U of Terminator 5′-Phosphate-Dependent Exonuclease (Lucigen) was added into the reaction supplied with 1× Exonuclease buffer A for 3 h at 30 °C to remove phosphorylated transcripts. The RNA enriched for 5′-capped transcripts is then purified with the RNA clean and concentrator-5 kit (Zymo Research). 30 U of Cap-Clip (CellScript) are added into the capped RNAs and incubated for 2 h at 37 °C to remove the cap. RNA is again purified with the RNA clean and Concentrator-5 kit. 10% of the treated RNA is saved as input, and 5 μg treated RNA is diluted and incubated with 5 μg of anti-m6A antibody (Abcam, ab151230) at 4 °C for 2 h. 100 μl protein G magnetic beads were added to pull down the m6A immunoprecipitation complex. After wash for three times, the bound RNA was eluted in 100 μl elution buffer and purified using RNA clean and concentrator-5 kit. Final libraries were amplified and were subjected to paired-end sequencing in the HT sequencing IGM Genomics core, UCSD.

### m^6^A-MeRIP and m^6^Am-Exo-MeRIP-seq analysis

Fastqc was used to perform quality control on sequencing data. And then, cutadapt was applied to remove adapters and trim reads. The pre-processed reads were aligned to human genome (built on Release 31 (GRCh38.p12) gencode) by STAR^53^. To call m^6^A peaks, MACS2^54^ (*q* value: 0.05, call-summit mode) was used based on its paired m^6^A-RIP/input data. The default peak range of m^6^A viewer is 200 nt and above 80% of peaks form MACS2 has range around 200 nt. To find the collection of common peaks for each group, peaks from biological replicates are filtered using an adjusted method based on the previously published paper^55^. Briefly, to define the common peaks, they should appear in at least two biological replicates, and peaks within 200 nt from each other are defined as one peak. These kept peaks together build the collection of common peaks for each group. To generate the peak distribution across chromosome region (including intron, CDS, intergenic region) and across gene region (3′UTR, 5′UTR and CDS) RSeQC^56^ and Guitar Plot^57^ were applied. The gene annotation used here is from GENCODE^58^. For further m^6^Am peak analysis, we extracted common peaks whose summit is in the 5′UTR region. The data visualization of these peaks is realized using bedtools^59^ and IGV^60,61^. To find potential m^6^Am peaks, peaks in 5′UTR region in the control group were compared with PCIF1 KO groups. In MeRIP seq, peaks in controlled groups but not in two PCIF1 KO groups (HIV− and HIV+) were considered as potential m^6^Am peaks. These potential m^6^Am peaks were then compared with peaks in HIV-infected groups to identify HIV-changed peaks. Potential peaks which have decreased peaks in control and HIV-infected groups are considered as m^6^Am peaks changed by HIV infection.

### Late RT-qPCR and Alu qPCR

To analyze the levels of late RT and integrated HIV DNA in control and PCIF1 KO cells, we used the methods developed by Bushman group^62^. Control or PCIF1 KO Jurkat cells were infected with HIVpp-luc at 10 or 48 h, genomic DNA was extracted and used for late RT using the primer MH531 and MH532. For Alu qPCR, 100 ng genomic DNA was firstly used to amplify the Alu-Gag segment with forward primer Alu (5′-GCCTCCCAAAGTGCTGGGATTACA-3′) and Reverse primer Gag (5′-GTTCCTGCTATGTCACTTCC-3′). Integrated HIV DNA into Alu hot pot were then quantified using qPCR using U5 primers and then normalized to genomic GAPDH expression.

### Immunoblotting

Cell lysates were separated in 4–12% pre-cast gel (Invitrogen) and transferred to PVDF membranes. Membranes were blocked with 5% FBS and primary antibodies were applied at 4 °C overnight or at RT for 2 h. After washing, membranes were incubated with HRP-conjugated secondary antibodies for 20 min (Pierce Fast Western Blotting Blot Kit) at room temperature. Finally, blots were washed three times and visualized using ECL substrate (Pierce Fast Western Blotting Blot Kit). Antibodies and dilutions were used as follows: anti-PCIF1 antibody at 1:1000 (Proteintech, 16082-1), anti-p24 antibody at 1:1000 (Abcam, ab9071), anti-p62 antibody at 1:1000 (Abcam, ab56416), anti-Vpr antibody at 1:1000 (Proteintech, 51143-1-AP), anti-Vpu antibody at 1:1000 (NIH AIDS Reagent Program, Cat# 969), anti-Nef antibody at 1:1000 (NIH AIDS Reagent Program, Cat# 1539), anti-Vif antibody at 1:1000 (NIH AIDS Reagent Program, Cat# 6460), anti GAPDH-HRP antibody at 1:5000 (Proteintech, HRP-60004), mouse anti-FLAG M2 antibody at 1:1000 (Sigma-Aldrich, F1804), anti-HDAC1 antibody at 1:1000 (Abcam, ab7028), anti ETS1 antibody at 1:2000 (Proteintech, 12118-1-AP), anti-GFP-tag antibody at 1:1000 (Proteintech, 50430-2-AP), HA antibody at 1:1000 (Santa-Cruz, sc-805). The density of western bands were quantified by ImageJ (https://imagej.nih.gov/ij/). GAPDH was used as a loading control.

### Quantitative real-time PCR (RT-qPCR)

Total RNA was extracted with TRIzol reagents (Invitrogen) according to the manufacturer’s instructions. 1 µg RNA was reverse transcribed using iScript cDNA Synthesis Kit (Bio-Rad). RT-qPCR was performed using 2× SYBR Green mix (Bio-Rad) and run on LightCycler480. PCR cycling conditions were 95 °C for 5 min followed by 45 cycles of 95 °C for 10 s, 60 °C for 10 s, and 72 °C for 10 s. All RT-qPCR primer sequences are listed in Supplementary Data 1.

### HIV detection using p24 ELISA, luciferase assay, or flow cytometry

For MT4 infected with LAI, supernatants were collected and viral p24 levels were detected using HIV-1 p24 Antigen ELISA Kit (RETROtek) following the manufacturers’ protocols. For cells infected with HIVpp-luc, cells were lysed and luciferase levels were measure using luciferase kit (Promege). For cells infected with HIVpp-GFP, cells were collected and fixed with fixing buffer (Biolegend) at RT for 10 min. After washing with PBS 2 times, cells were separated into single cell using cell strainer. About 1 million cells were applied onto flow cytometry to detect GFP expression.

### Immunoprecipitation

To identify Vpr interactions with PCIF1, 3 million HeLa cells were seeded into a dish one day before transfection. 3 μg plvx-PCIF and 9 μg Flag-Vpr vectors were co-transfected into cells using lipofectamine 3000 according to its instruction. To identify whether PCIF1 could be ubiquitinated by Vpr, Vpr and Vpr-Q65R stably expressed HeLa cell line was created. 3 million Vpr, Vpr-Q65R, or control HeLa cells were seeded into a dish one day before transfection. 3 μg plvx-PCIF1 and 5ug HA-Ub vectors were co-transfected into cells using lipofectamine 3000 according to its instruction. Two days later, cells were lysed in IP lysis buffer (ThermoFisher) supplemented with protease inhibitor and pre-cleared with 20 μl magnetic protein G beads (CST) at 4 °C for 2 h. And then 1 mg cell lysate was incubated with either 4 μg mouse IgG antibody (Invitrogen, 02-6502) or anti-Flag M2 antibody (Sigma-Aldrich, F1804) at 4 °C overnight. The next day, 40 μl BSA pre-blocked magnetic protein G beads were added and incubated for 2 h at 4 °C. After washing three times, 10 μl protein loading buffer (Invitrogen) were directly added into the beads and protein was denatured at 95 °C for 10 min. Beads were then removed using magnet and samples were applied to the western blotting blot to detect the expression of target proteins. The reciprocal HA-IP was also performed in the same samples to determine the HA ubiquitin protein is exactly PCIF1, 1 mg cell lysate was incubated with HA-conjugated beads (sigma) at 4 °C for 2 h

### Chromatin immunoprecipitation of ETS1

To analyze ETS1-HIV proper interactions, ETS1-CHIP was performed. Briefly, MT4 cells (2 × 10^7^) were infected with HIV-1 at an MOI of 0.2 for 3 days and then harvested and fixed with 1% formaldehyde for 10 min at room temperature. The reaction was quenched using glycine at a final concentration of 125 mM. Fixed cells were lysed and sonicated to around 400 bp fragment. 30 µg chromatin were pre-cleared and incubated with 4 µg control rabbit IgG antibody (ab4729; Abcam) or anti-ETS1 antibody (#14069, Cell signaling technology). CHIP primer of c-myc was purchased from CST(#14905).

### Nuclear and cytoplasmic fractionation

Cells were washed with PBS, and suspended in cytoplasm lysis buffer (10 mM Tris-Cl pH7, 1.5 mM MgCl2, 10 mM KCl, 0.5 mM DTT, 1 mM PMSF, 0.25% NP-40) supplemented with protease inhibitor for 10 min at 4 °C and were centrifuged at 2500×*g* for 15 min to pellet the nuclear. Cytoplasmic RNA and protein were kept in the supernatant. The nuclear pellet was washed twice with the cytoplasm lysis buffer. And then suspended in cytoplasm lysis buffer (25 mM Tris-Cl pH7, 0.5% NP-40, 150 mM KCl, 0.5 mM DTT, 1 mM PMSF) supplemented with protease inhibitor followed by centrifugation at 16,000×*g* for 10 min to pellet the debris and keep the nuclear supernatant. Then, TRIzol was added to cells, cytoplasmic or nuclear supernatant to purify RNA. Results were presented as a percentage of RNA relative to whole-cell input (set as 100%). For detection of PCIF1, Vpr, GAPDH (cytoplasm protein control), or HDAC1 (nuclear control) protein distribution, protein in the cytoplasmic or nuclear supernatant was denatured and 25 μg proteins were applied to western blotting.

### PCIF1-cross-linking and RNA immunoprecipitation (CLIP)

RNA immunoprecipitation was performed in Flag-PCIF1 expressing MT4 cells. 40 million cells were collected, and then washed with PBS. Cells were suspended with 10 ml of cold PBS and irradiated once with 400 mJ/cm^2^ at 254 nm. MT4 cells were then harvested and nuclear extracts were isolated followed the protocol described above. Nuclear extracts were sonicated and 1 mg of nuclear extracts was incubated with 4 µg of IgG antibody (Invitrogen, 02-6502) or anti-Flag M2 antibody (Sigma-Aldrich, F1804) at 4 °C overnight. The following day, 40 μl BSA pre-blocked magnetic protein G beads were added into the protein-antibody complex and incubated for 2 h at 4 °C. After washing three times, beads were suspended in 100 μl PBS, followed by 20 U DNase I (New England Biolabs) digestion at 37 °C for 15 min. Protein was digested by incubation with 50 μg of Proteinase K (New England Biolabs) at 37 °C for 15 min. Finally, RNAs were recovered by TRIzol, reversed transcribed, and detected by RT-qPCR.

### RNA half-life measurement

To determine RNA stability of the m^6^Am genes. One million control and PCIF1 KO cells were treated with 5 μg/ml Actinomycin D (ActD) to block gene transcription. Cells were collected at 0, 2, 4, 8, 16, and 24 h after actinomycin D (ActD) treatment. RNA was extracted using TRIzol. 1 µg RNA was reverse transcribed and RNA expressions were detected by RT-qPCR and shown as percentage relative to mock treatment. GAPDH expression was not changed in PCIF1 cells and was shown as a negative control. Linear regression analysis was carried out to calculate the half-life of mRNA.

### Statistical analysis

Details of the statistical analysis were shown in the figure legend. The comparisons were performed using GraphPad 8. The difference between two groups was calculated by Student’s *t*-test. Statistically, significance results were determined when *p* < 0.05. *p < 0.05, **p < 0.01, ***p < 0.001, ****p < 0.0001. Data are presented as the mean with standard deviations as indicated.

### Reporting summary

Further information on research design is available in the Nature Research Reporting Summary linked to this article.

## Supplementary information

Supplementary Information

Description of Additional Supplementary Files

Supplementary Data 1

Supplementary Data 2

Supplementary Data 3

Supplementary Data 4

Reporting Summary

## Data Availability

The accession number for the m^6^A-MeRIP-seq sequencing data reported in this paper is NCBI GEO: GSE154035. The accession number for the m^6^Am-Exo-Seq sequencing data reported in this paper is NCBI GEO: GSE171800. The raw data for LC/MS quantification curves of modified RNA and uncropped versions of western blots are provided in the Source Data file. Source data are provided with this paper.

## Source data

Source Data

## Code availability

The coding for m^6^A-MeRIP and m^6^Am-Exo-Seq analysis is deposited at Github: https://github.com/KylinKang-bme/m6Am_code, which is also available at zenodo: 10.5281/zenodo.5173217^63^.
