# Supplementary material for: HIV reprograms host m6Am RNA methylome by viral Vpr protein-mediated degradation of PCIF1
Source: Nat Commun. 2021 Sep 20;12:5543. doi: 10.1038/s41467-021-25683-4 (PMC8452764; doi:10.1038/s41467-021-25683-4)
Supplement: Supplementary file 1 — Supplementary Information [file 41467_2021_25683_MOESM1_ESM.pdf]

Supplementary Information

HIV reprograms host m^{6}Am RNA methylome by viral Vpr protein-mediated degradation of PCIF1

Qiong Zhang, Yuqi Kang, Shaobo Wang, Gwendolyn Michelle Gonzalez, Wanyu Li, Hui Hui, Yinsheng Wang, and Tariq M. Rana

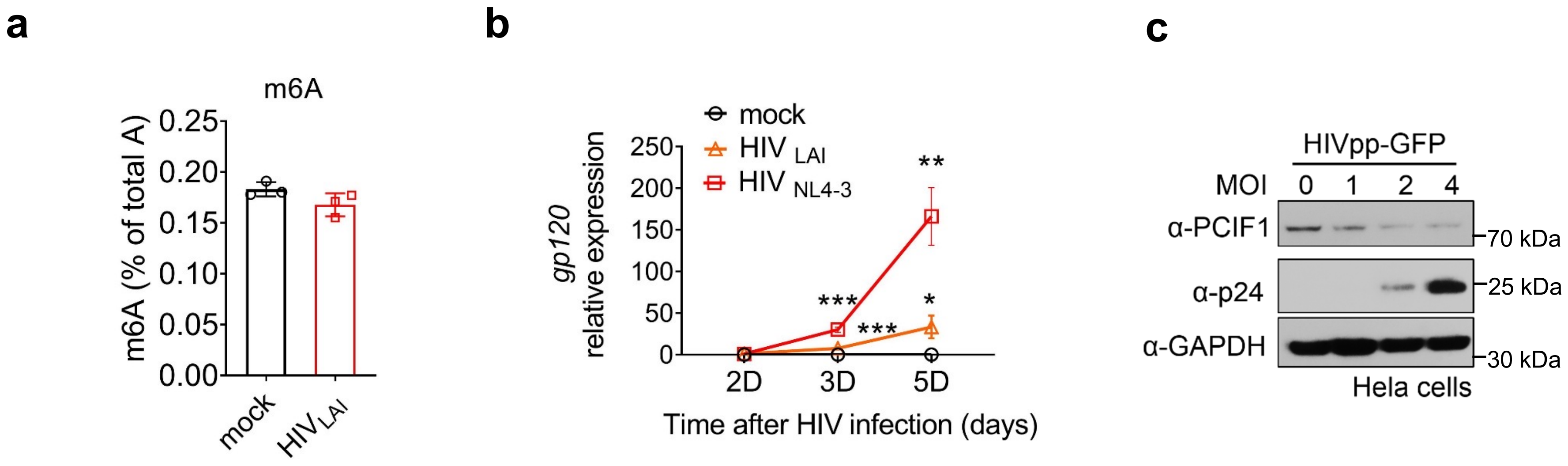

Supplementary Figure 1. HIV infection dose-dependently decreases PCIF1 in T cells and HeLa cells

(a) m^6A mRNA of cellular mRNA is not significantly altered by HIV infection at MOI 0.4 in MT4 cells. m^6A in cellular mRNA was detected by LC-MS/MS in the same parallel of samples as in Fig 1a. n = 3 independent biological experiments. Two-sided *t* test. Mean ± SD.

(b) Replication kinetics of HIV in MT4 cells. MT4 cells were infected with HIV_{NL4-3} (MOI=2) and HIV_{LAI} (MOI =0.4). Replication of HIV was detected by measuring *gp120* mRNA expressions at different time points to obtain the replication kinetics of HIV in MT4 cells. n = 3 independent biological experiments. Two-sided *t* test. Mean ± SD. ***p=0.0001, **p=0.0012, ***p=0.00079, *p=0.015.

(c) PCIF1 protein is degraded dose dependently by HIV infection in HeLa cells. Western blotting blot of PCIF1 and p24 in HeLa cells infected with HIV pseudovirus (HIVpp-GFP) at increasing MOI (MOI = 1, 2, and 4) for 2 days. The results of c were representative images of at least three independent experiments.

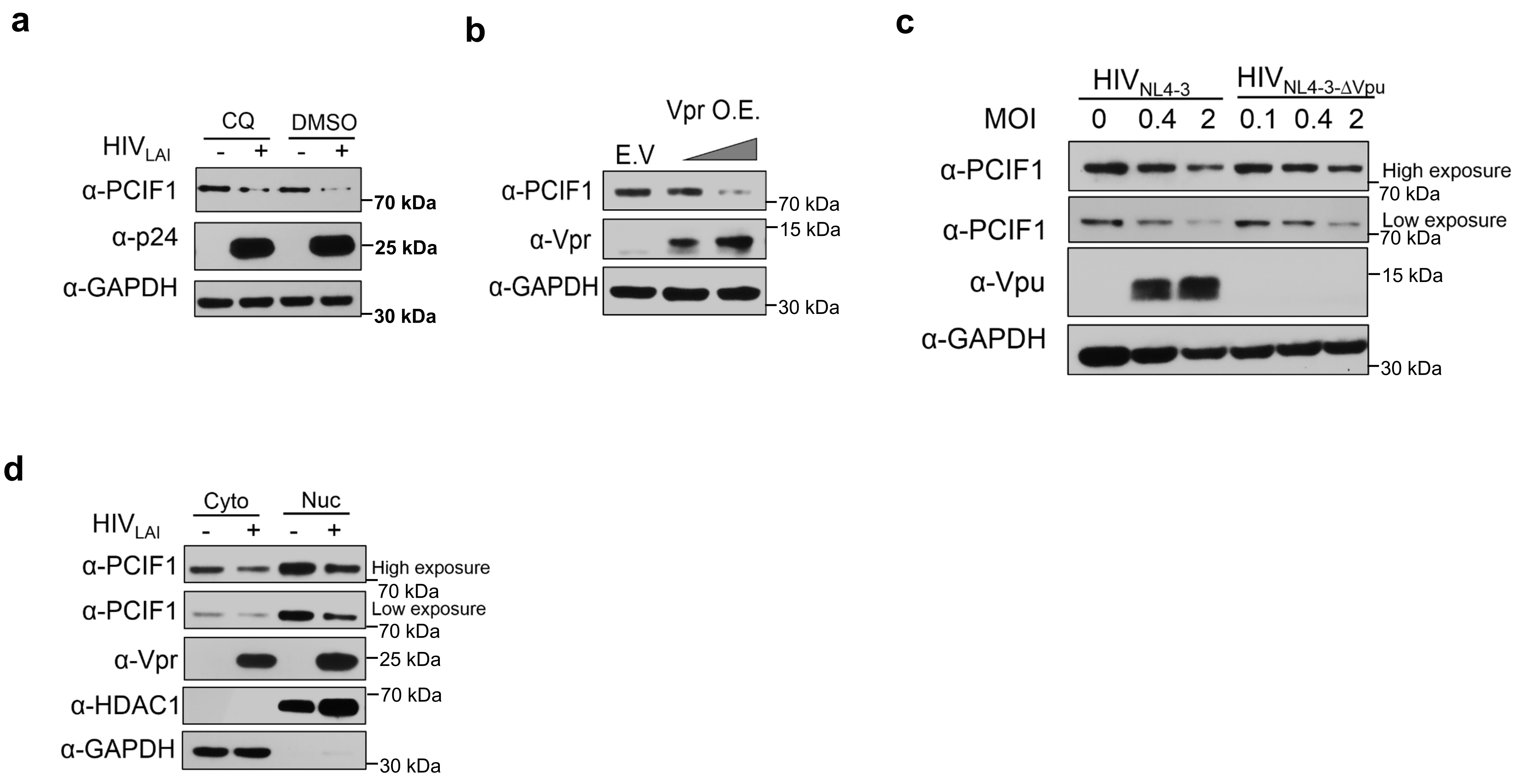

Supplementary Figure 2. PCIF1 is degraded by Vpr.

(a) PCIF1 degradation by HIV is not affected by lysosome inhibitor. MT4 cells were infected with HIV_{LAI} for 3 day at an MOI of 0.4. Cells were incubated with DMSO or Chloroquine (CQ,10 μM) at 1 day before lysing cells. PCIF1, p24 protein levels were detected by Western blotting.

(b) PCIF1 is down-regulated by Vpr in a dose dependent manner. HeLa cells were transfected with 1μg empty vector or two doses of Vpr overexpression vector (0.5 μg or 1μg) for 3 days. PCIF1 and Vpr expressions were analyzed.

(c) Vpu deleted virus does not rescue PCIF1 expression. MT4 was infected with NL4-3 or Vpu deleted virus at indicated MOIs for 3 days. PCIF1, p24, and Vpu expressions were then detected.

(d) PCIF1 is mainly distributed and degraded in the nucleus. MT4 cells were infected with HIV_{LAI} for 3 days (MO=0.4) and then cytoplasm and nuclear proteins were separated. The expressions of PCIF1, Vpr, GAPDH, and HDAC1 in the nucleus or cytoplasm were detected.

The results of a-d were representative images of at least three independent experiments.

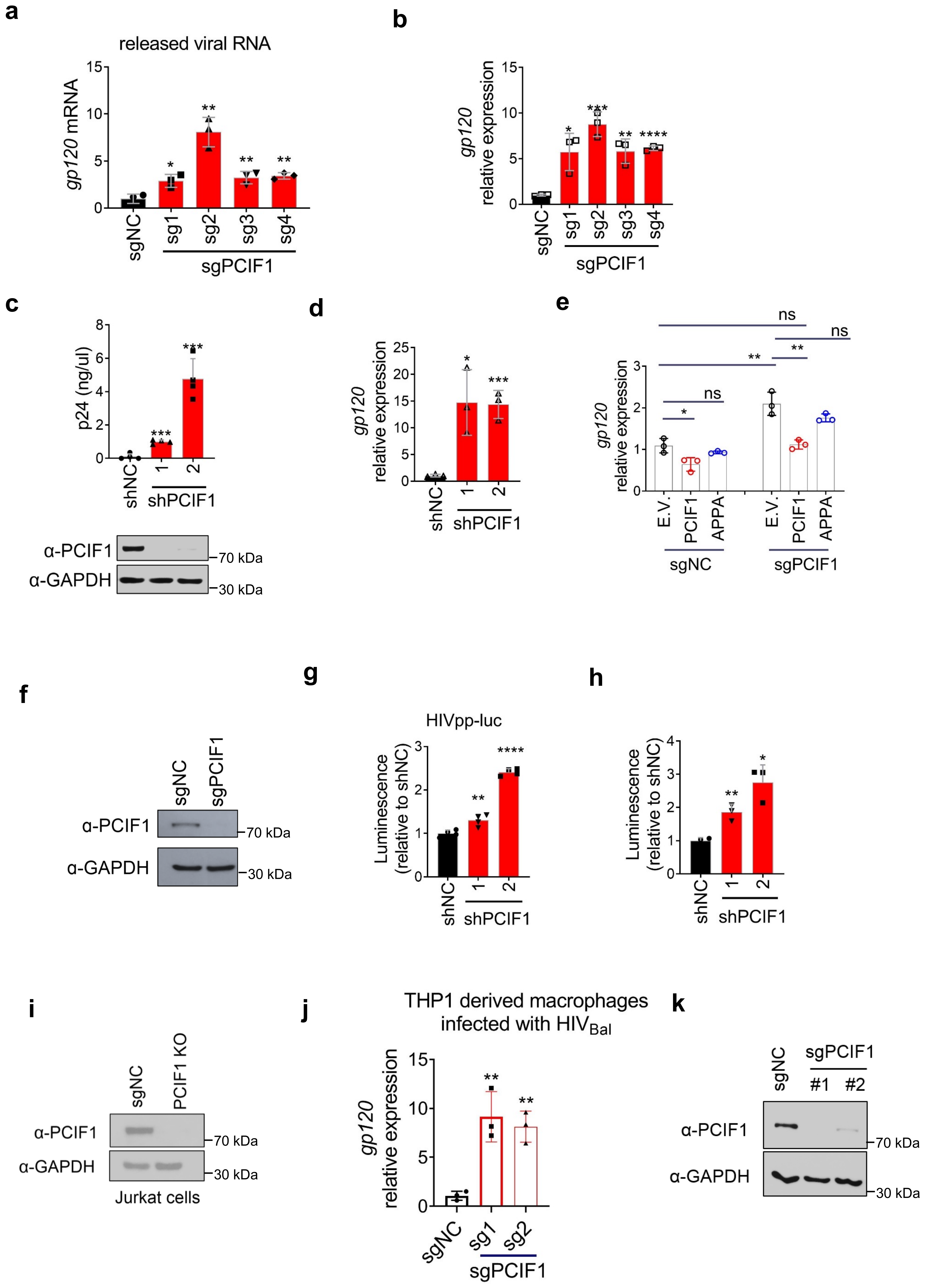

a
released viral RNA
gp120 mRNA
sgNC
sg1
sg2
sg3
sg4
sgPCIF1
b
gp120 relative expression
sgNC
sg1
sg2
sg3
sg4
sgPCIF1
c
p24 (ng/ul)
shNC
1
2
shPCIF1
α-PCIF1
α-GAPDH
70 kDa
30 kDa
d
gp120 relative expression
shNC
1
2
shPCIF1
e
ns
ns
gp120 relative expression
E.V.
PCIF1
APPA
E.V.
PCIF1
APPA
sgNC
sgPCIF1
f
sgNC
sgPCIF1
α-PCIF1
α-GAPDH
70 kDa
30 kDa
g
HIVpp-luc
Luminescence (relative to shNC)
shNC
1
2
shPCIF1
h
Luminescence (relative to shNC)
shNC
1
2
shPCIF1
i
sgNC
PCIF1 KO
α-PCIF1
α-GAPDH
70 kDa
30 kDa
Jurkat cells
j
THP1 derived macrophages infected with HIVBal
gp120 relative expression
sgNC
sg1
sg2
sgPCIF1
k
sgNC
sgPCIF1
#1
#2
α-PCIF1
α-GAPDH
70 kDa
30 kDa

Supplementary Figure 3. PCIF1 inhibits HIV infection.

(a, b) KO of PCIF1 promotes HIV replication in MT4 cells. Control and PCIF1 KO cells were treated as in 2b. Release HIV RNA (a) and cellular HIV RNA (b) were analyzed to quantify HIV replication using RT-qPCR. (a) *p=0.018, **p=0.0017, **p=0.0096, **p=0.0022. (b) *p=0.016, ***p=0.00058, **p=0.0034, ****p=4.98673E-06.

(c, d) Knock-down of PCIF1 increases HIV replication in MT4 cells. Levels of p24 and PCIF1 protein (c), and *gp120* mRNA expressions (d) were quantified in control shRNA (shNC) or PCIF1 knock-down shRNA (shPCIF1 #1-2) transduced MT4 cells infected with HIV_{LAI} at an MOI of 0.01 for 3 days. p24 was analyzed by ELISA. (c) ***p=0.00013, ***p=0.00028. (d) *p=0.018, ***p=0.00096.

(e) PCIF1 restricts HIV infection and is dependent on PCIF1 methyltransferase activity. HIV *gp120* RNA expression was detected in cells as treated in Fig. 2c. *p=0.03, **p=0.006, **p=0.0048, ns, not significant.

(f) CRISPR-mediated knock-out of PCIF1 was detected in sgNC and sgPCIF1 transduced 293 FT cells by Western blotting.

(g, h) KD of PCIF1 increases single cycle HIV replication. Control and PCIF1 KD MT4 cells (g) or Jurkat cells (h) were infected with HIV single cycle virus (HIVpp-luc) at an MOI of 0.2. Luciferase reporter expression was measured at day 2 post infection. (g) **p=0.0078, ****p=2.86323E-07. (h) **p=0.0046, **p=0.0048.

(i) PCIF1 knock-out Jurkat T cell colony was chosen and shown to be depleted of PCIF1 expression by Western blotting.

(j and k) KO of PCIF1 increases HIV replication in macrophages. Control sgRNA or two PCIF1 specific sgRNAs were transduced into THP1 macrophages for 1 week to knock out PCIF1 expression. THP1 cells were then derived into macrophages and infected with HIV_{Bal} (MOI=1, 3 days). Levels of *gp120* mRNA expressions were quantified in control or PCIF1 knock out cells using RT-qPCR. **p=0.0059, **p=0.0018. (j). PCIF1 KO efficiency is detected (k).

All data are represented as mean ± SD and analyzed by two-sided *t* test in b-g. n = 3 (a-b, d-e, h, j), or 4 (c, g) independent biological experiments. Similar results were obtained from three independent experiments in c, f, i, k.

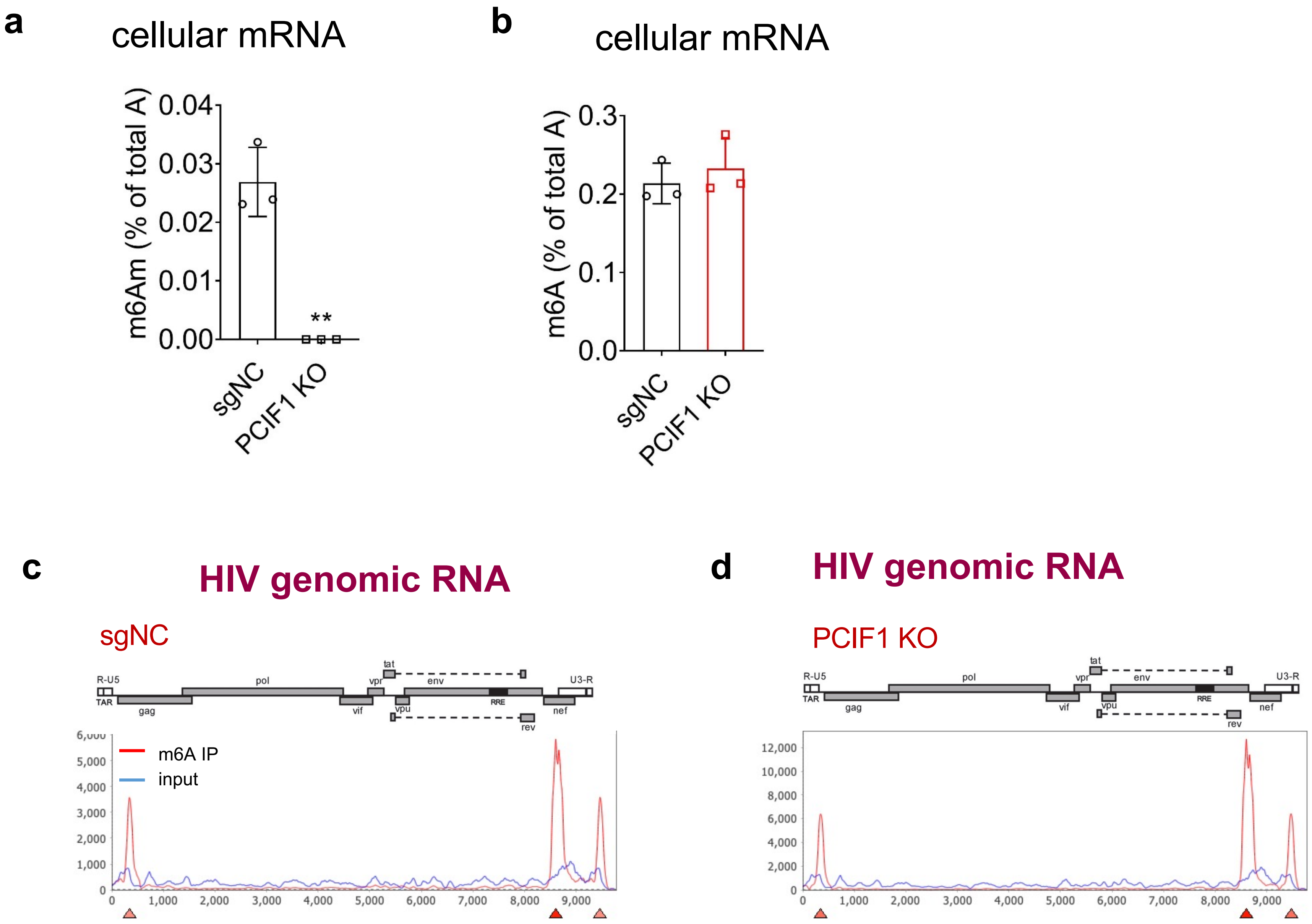

Supplementary Figure 4. HIV genomic RNA is not modified by m^{6}Am.

(a, b) PCIF1 KO completely depletes the m^{6}Am modification of cellular mRNA. m^{6}Am (a) or m^{6}A (b) levels in cellular mRNA was detected by LC-MS/MS in control and PCIF1 KO cells. ***p= 0.0014, n=3 independent biological experiments. Two-sided t test. Mean ± SD.

(c, d) m^{6}A-MeRIP peak of HIV genomic RNA is not altered in PCIF1 KO cells. Genome tracks of HIV genomic RNA were plotted with called m^{6}A peaks in control or PCIF1 KO cells infected with HIV_{LAI}. One representative of three experiments are shown.

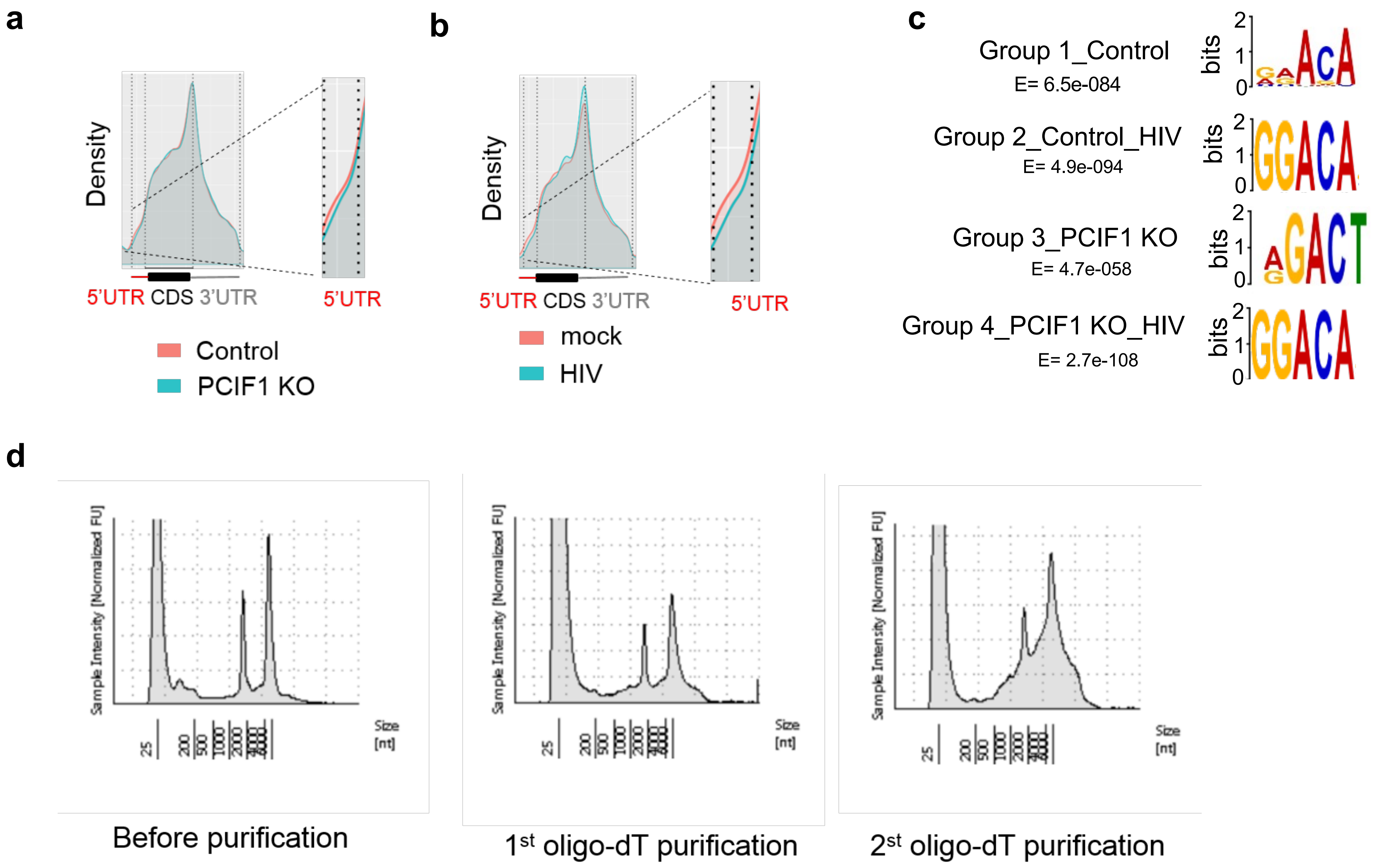

Supplementary Figure 5. Identification of m^{6}Am modified cellular genes altered by HIV infection using m^{6}A-MeRIP sequencing

(a) m^{6}A-MeRIP peaks in the 5'UTR are decreased in PCIF1 KO cells. Metagene analysis of m^{6}A-MeRIP reads in control or PCIF1 KO Jurkat cells.

(b) m^{6}A-MeRIP peaks in the 5'UTR are reduced in HIV infected cells. Metagene analysis of m6A- MeRIP reads in Jurkat cell mock-infected or infected with HIV_{LAI} at an MOI of 4. Enlarged sections in a and b shows the m^{6}A peaks in the annotated 5'UTR regions.

(c) DREME motif search to identify conserved motif of m^{6}A-MeRIP reads in control Jurkat cells or PCIF1 KO Jurkat cells mock-infected or infected with HIV_{LAI} at an MOI of 4.

(d) The bioanalyzer plots show the mRNA samples prepared for sequencing, including total RNA before purification, and mRNA after one or two rounds of oligo-dT purification.

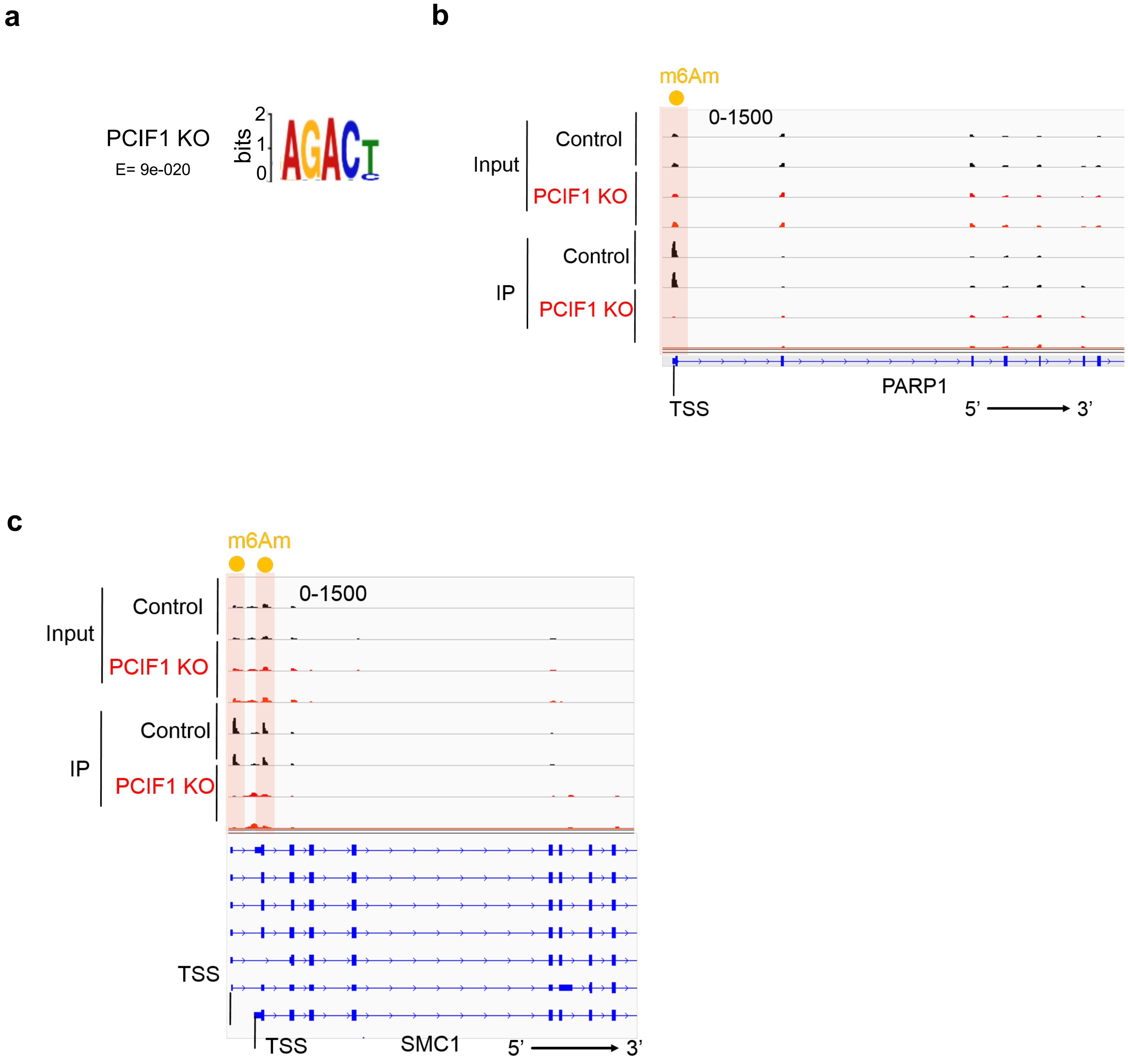

Supplementary Figure 6. Representative m^{6}Am modified cellular genes in T cells using m^{6}Am-Exo-MeRIP sequencing

(a) DREME motif search to identify conserved motif of m^{6}A reads in PCIF1 KO Jurkat cells.

(b and c) m^{6}Am-Exo-MeRIP peaks of PARP1 (b) and SMAC1 (c) genes in the 5’ UTR were decreased in PCIF1 KO cells compared to control cells.

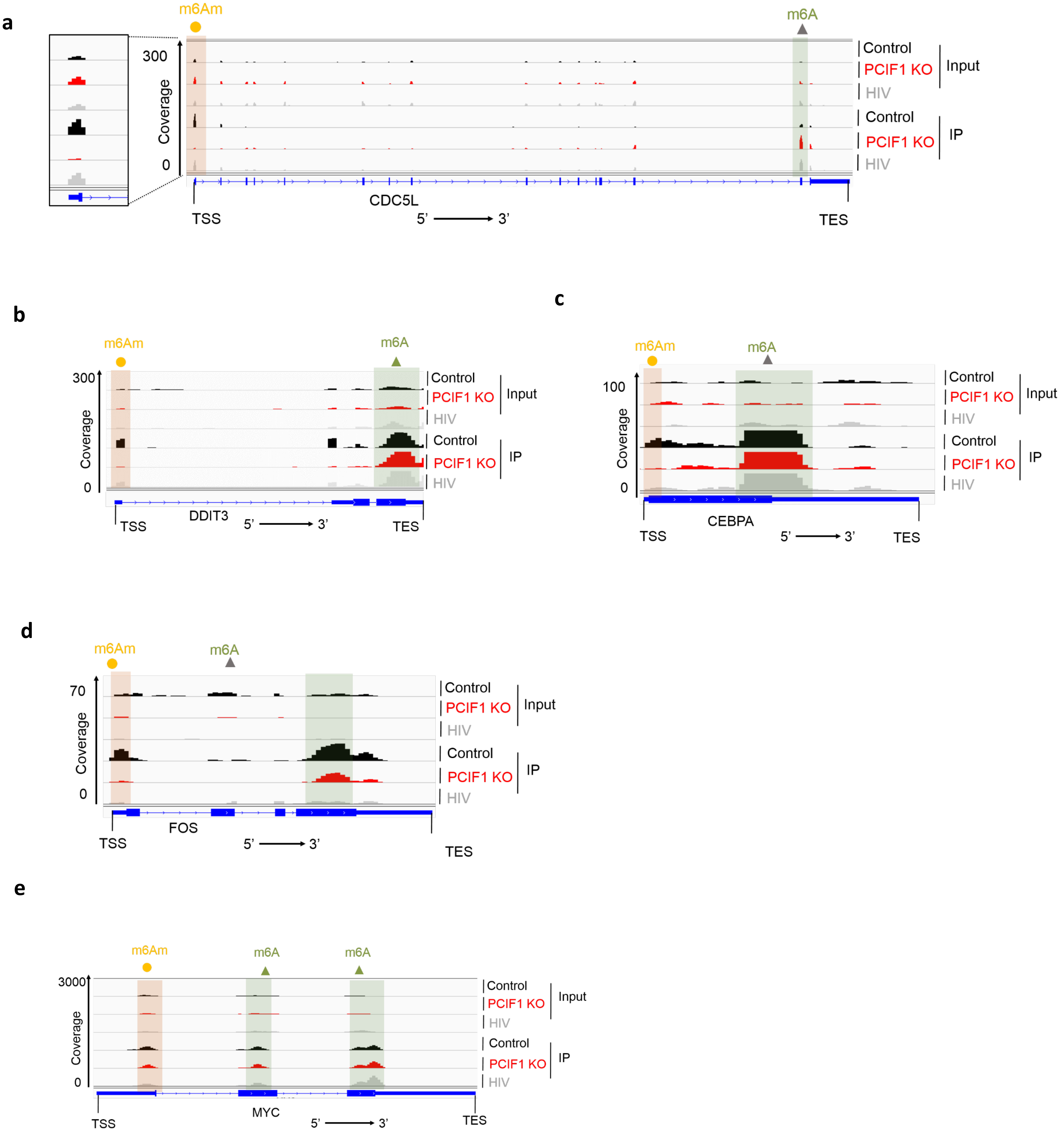

Supplementary Figure 7. Representative m^{6}Am modified cellular genes that are changed by HIV using m^{6}Am-Exo-MeRIP sequencing

m^{6}A-Exo-MeRIP peaks of CDC5L (a), DDIT3 (b), CEBPA (c), FOS (d), and MYC (e) genes in the 5' UTR were decreased in PCIF1 KO cells and HIV infected cells. One representative of two experiments is shown.

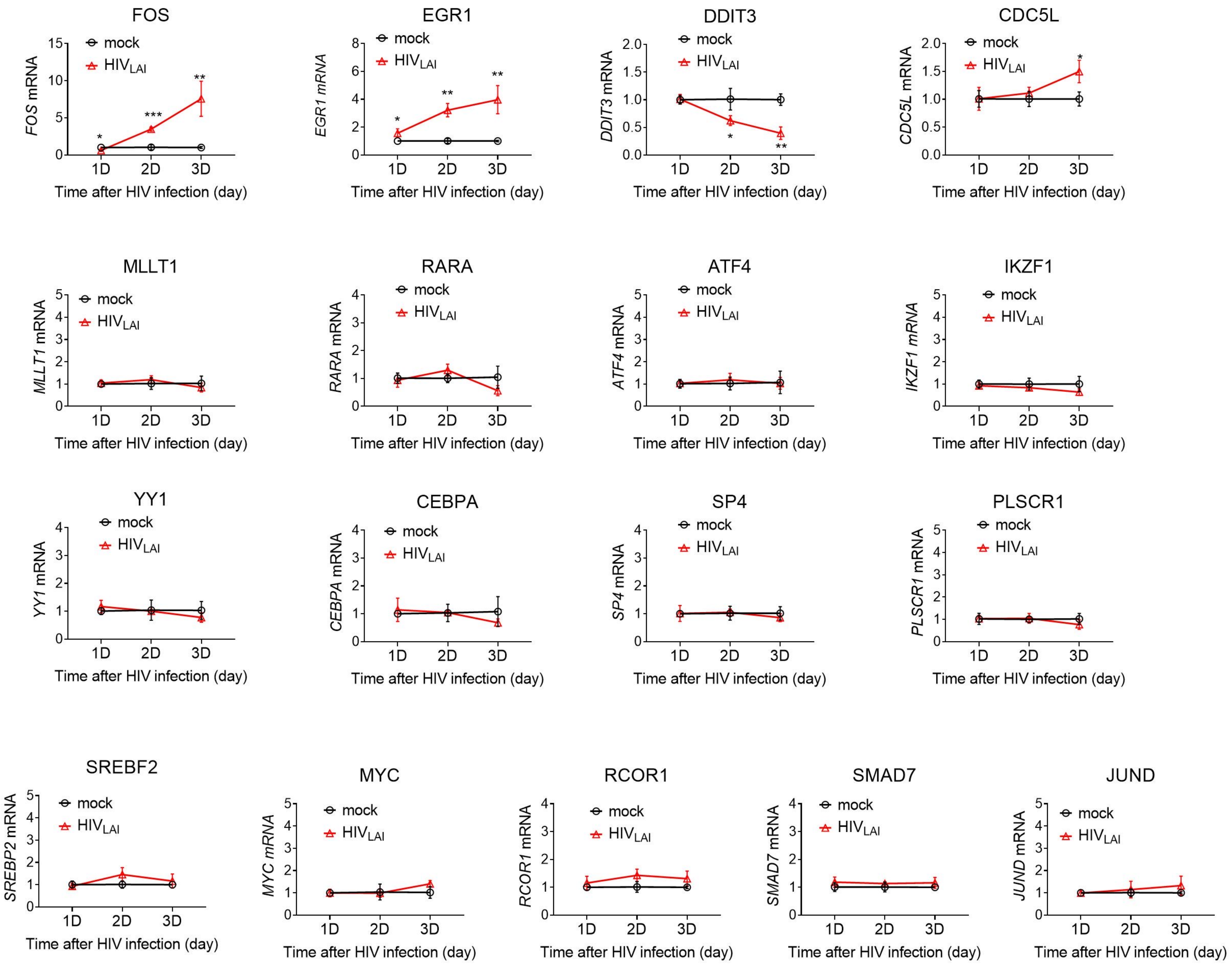

Supplementary Figure 8. Analysis of RNA levels of m^{6}Am target genes.

Kinetics of 17 m^{6}Am transcripts during HIV infection. The indicated mRNA expressions were quantified in MT4 cells infected with HIV$_{LAI}$ at MOI 0.4 for 1, 2, or 3 days. Two-sided *t* test. Mean ± SD, n=3 biological independent experiments. FOS: **p*=0.035, ****p*=0.00067, ***p*=0.009. EGR1: **p*=0.04, ***p*=0.0018, ***p*=0.007. DDIT3: **p*=0.035, ***p*=0.0023. DDIT3: **p*=0.023.

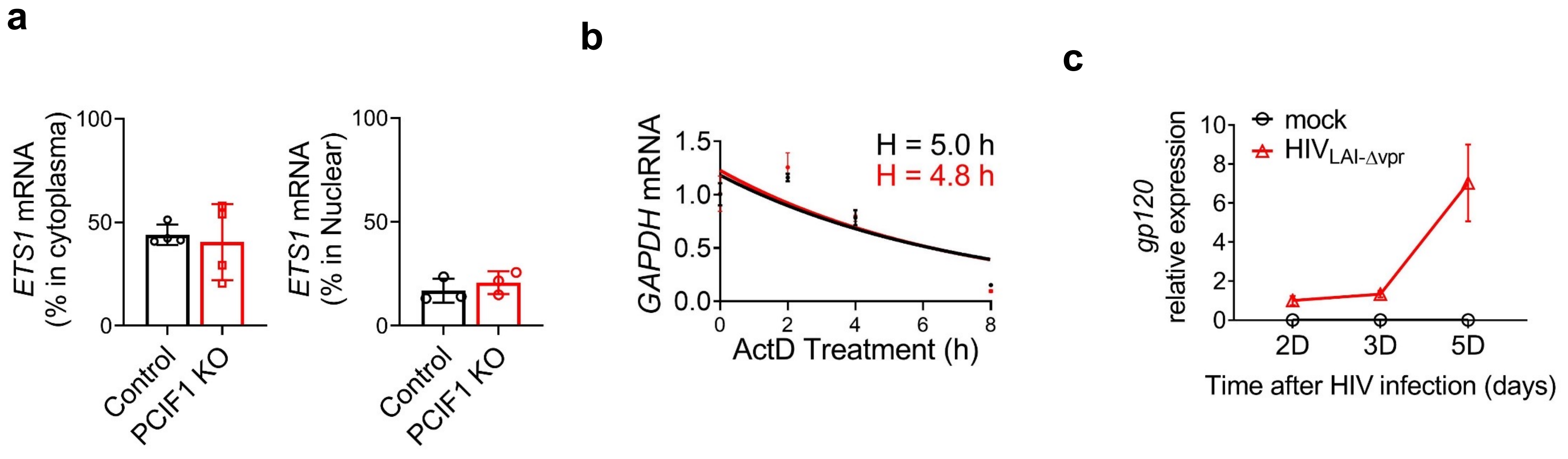

Supplementary Figure 9. Analysis of RNA translocation, stability and translation levels in 18 targeted m^6Am genes.

(a) The export of *ETS1* mRNA is not changed in the PCIF1 KO cells. The cytoplasm fraction (left) or the nucleus fraction (right) was isolated from control or the PCIF1 KO Jurkat cells. The percentage of cytoplasm or nucleus RNA relative to whole cellular RNA is shown. Two-sided *t* test. Mean ± SD, n=3 independent biological experiments.

(b) The decay rate of GAPDH mRNA in control or PCIF KO cells. Control or PCIF1 KO Jurkat cells were treated with ActD and GAPDH RNA were quantified by RT-qPCR. Two-sided *t* test. Mean ± SD, n=3 independent biological experiments.

(c) Replication kinetics of $HIV_{LAI\text{-}\Delta Vpr}$ in MT4 cells. MT4 cells were infected with $HIV_{LAI\text{-}\Delta Vpr}$ (MOI =0.4). Replication of HIV was detected by measuring *gp120* mRNA expressions at different time points to obtain the replication kinetics of HIV in MT4 cells. Two-sided *t* test. Mean ± SD, n=3 independent biological experiments.

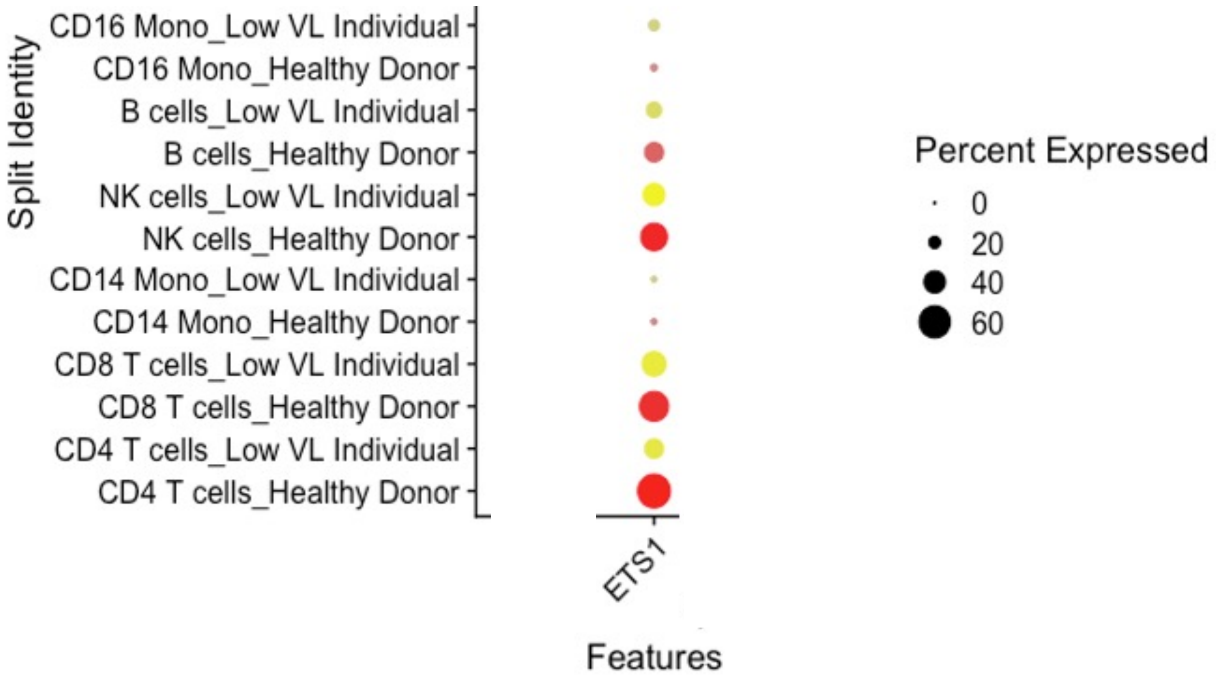

Supplementary Figure 10. Expression of ETS1 in HIV infected individuals.

ETS1 mRNA is reduced in CD4 T cells of HIV infected individuals. Expression in six of the cell clusters (including CD4 T cells, CD8 T cells, CD14 monocytes, NK cells, B cells, and CD16 monocytes) in healthy donors and HIV-infected individuals. The color intensity shows the average expression levels and the circle size indicates the percentage of expressing cells in each cluster.

Supplementary Methods: LC/MS quantification of modified RNA

LC-MS/MS/MS quantification of modified ribonucleosides

Enzymatic digestion was preformed using previously published method.[1] Briefly, 50 ng of mRNA was incubated at 37°C for 2 hr with 0.5 unit of nuclease P1 (NP1) in 25 µL buffer containing 25 mM NaCl and 2.5 mM $ZnCl_2$. To the mixture were subsequently added 0.25 unit of alkaline phosphatase (CIP) and 3 µL of 1 M NH_4HCO_3 buffer. After incubating at 37°C for an additional 2 hr, the digestion mixture was dried by a Speed-vac and reconstituted in 100 µL of H_2O. Profiling and quantification of modified ribonucleoside LC-MS/MS and MS/MS/MS were conducted following previously published procedures with minor modifications.[2] In short, to 10 ng of digested RNA sample were added 8.00 pmol of [$^{13}C_5$]-rA and 42.5 fmol of [D_3]-m^6A. The enzymes in the digestion mixture were removed by chloroform extraction. The aqueous layer was dried and reconstituted in a 100 µL solution of H_2O/CH_3CN (1/9, v/v). After incubating at -20°C for 20 min, the mixture was centrifuged and the supernatant was collected, and dried by Speed-vac.
LC-MS/MS and MS/MS/MS experiments were performed on an LTQ-Orbitrap Velos mass spectrometer coupled with an EASY-nLC II (Thermo Fisher Scientific, San Jose, CA). Samples were loaded onto a 5 cm in-house packed Magic C18-AQ pre-column (150 µm i.d, 5 µm in particle size, and 100 Å in pore size; Michrom BioResources, Auburn, CA). Analytes were resolved on a 20 cm Magic C18-AQ analytical column (75 µm i.d.) at a flow rate of 300 nL/min. The gradient employed was 0-40% B in 20 min, 40-90% B in 5 min, and finally 5 min at 90% B using 0.1% (v/v) formic acid in water and 0.1% (v/v) formic acid in acetonitrile as mobile phases A and B respectively. The mass spectrometer was operated in the positive-ion mode with the electrospray, capillary, and tube lens voltages being 2.0 kV, 12 V, and 100 V, respectively. The detailed parameters for precursor ion selection and collisional energy employed for MS/MS and MS/MS/MS analyses are listed in Table S1. Calibration curves were constructed by analyzing mixtures of different amounts of unlabeled standards with fixed amounts of stable isotope-labeled standards under the same LC-MS/MS and MS/MS/MS conditions. In particular, [$^{13}C_5$]-rA was employed as the internal standard for quantifying rA, and [D_3]-m^6A was used for quantifying [D_3]-m^6A and m^6A_m. Representative selected-ion chromatograms and calibration details are presented below.

References

1. Wang, X.; Lu, Z.; Gomez, A.; Hon, G. C.; Yue, Y.; Han, D.; Fu, Y.; Parisien, M.; Dai, Q.; Jia, G.; Ren, B.; Pan, T.; He, C., *Nature* **2014,** *505* (7481), 117-20.
2. Fu, L.; Amato, N. J.; Wang, P.; McGowan, S. J.; Niedernhofer, L. J.; Wang, Y., *Anal. Chem.* **2015,** *87ha* (15), 7653-7659.

	Transitions	Isolation Width		NCE	
		MS^2	MS^3	MS^2	MS^3
rA (MS/MS)	268 → 136	3	2	35	
[$^{13}C_5$]-rA	273 → 136	3	2	35	
m^6A	282 → 150 → 94	3	2	43	37
[D_3]-m^6A	285 → 150 → 94	3	2	43	37
m^6A_m	296 → 150 → 94	3	2	37	35

Supplementary Table 1. Transitions employed for the quantifications of 2'-deoxyadenosine (rA), *N*^{6}-methyadenosine (m^6A) and *N*^{6},2′-O-dimethyladenosine (m^6A_m). Shown are also the isolation widths for precursor ions and the normalized collision energy (NCE) values employed in MS/MS and MS/MS/MS experiments.

		Replicate 1	Replicate 2	Replicate 3	Mean ± S.D.
m^6A level (modifications per 100 rAs)	MT4-mock	0.180	0.191	0.178	0.183 ± 0.007
	MT4-LAI	0.171	0.155	0.177	0.168 ± 0.012
m^6A_m level (modifications per 100 rAs)	MT4-mock	0.027	0.028	0.029	0.028 ± 0.001
	MT4-LAI	0.017	0.018	0.020	0.019 ± 0.001

Supplementary Table 2. The levels of m^6A and m^6A_m in mRNA samples isolated from MT4 cells that are mock-treated (MT4-mock) or transfected with HIV_{LAI} (MT4-LAI). The data represent the results from LC-MS/MS/MS measurements of three biological replicates, where each replicate was measured by LC-MS/MS twice. “S.D.” represents standard deviation.

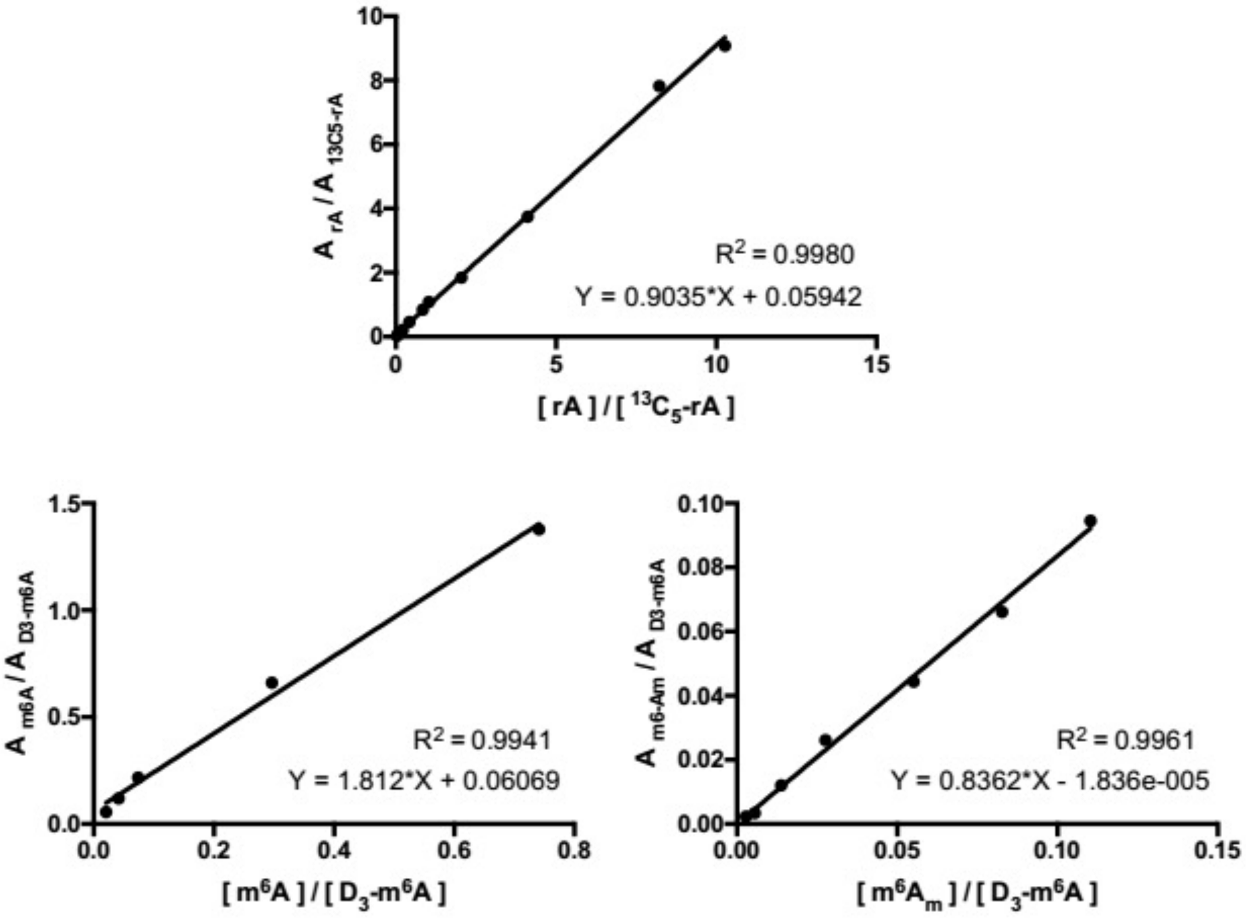

Supplementary Figure 11. Calibration curves for the quantifications of adenosine (rA), *N*^{6}-methyladenosine (m^{6}A), and *N*^{6},2′-*O*-dimethyladenosine (m^{6}A$_m$). For rA calibration, 3.3 pmol of [$^{13}C_5$]-rA was mixed with unlabeled rA standard ranging from 105.8 fmol to 32 pmol, and the resulting samples were analyzed by LC-MS/MS. Calibration curves for m^{6}A$_m$ and m^{6}A were generated by LC-MS/MS/MS analysis of mixtures containing 85 fmol of [D$_3$]-m^{6}A internal standard and unlabeled standards of m^{6}A and m^{6}A$_m$ ranging from 1.8 to 63 fmol and from 0.45 to 9.4 fmol, respectively.

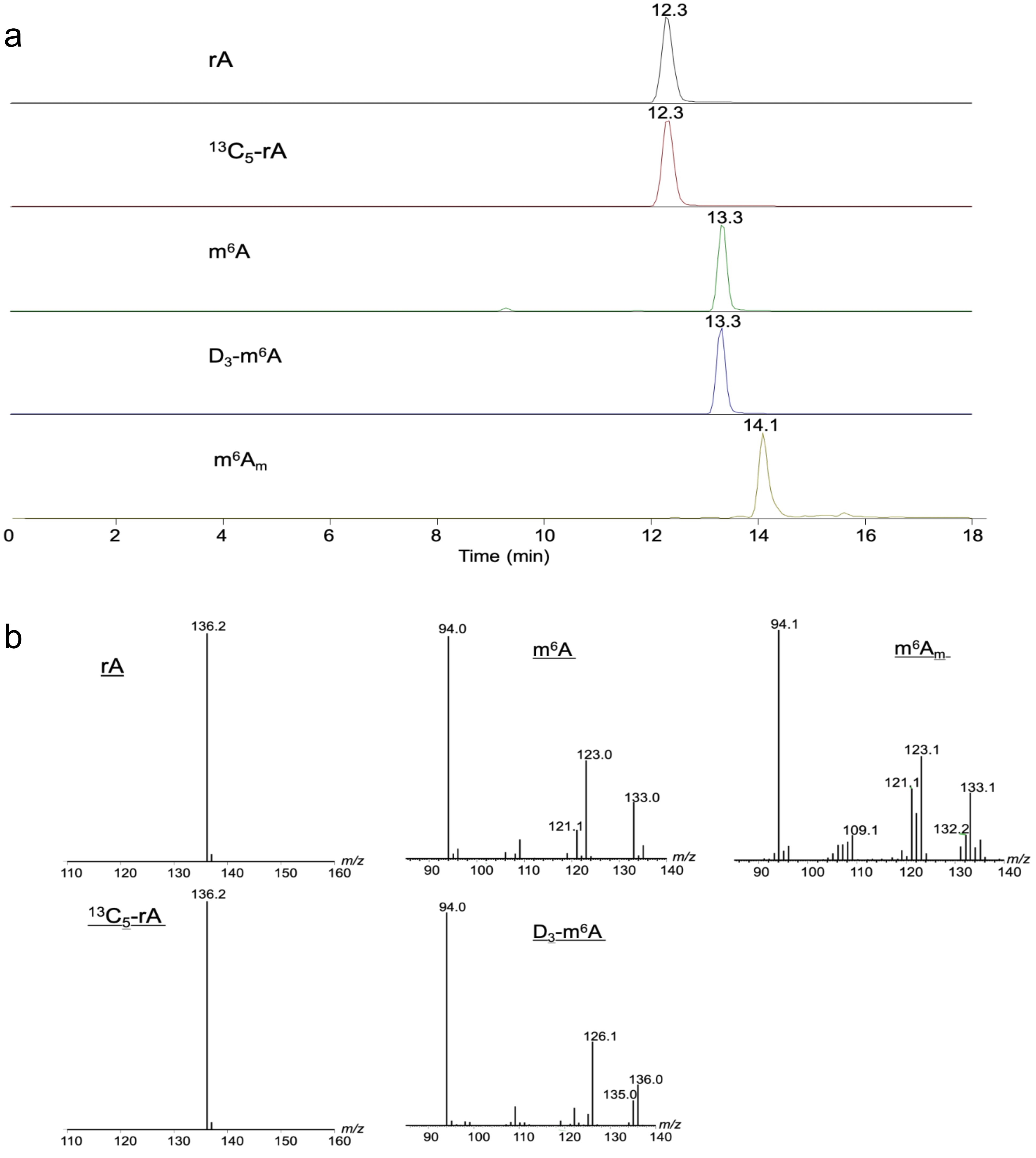

Supplementary Figure 12. Representative selected-ion chromatograms (SICs) (a) and the corresponding tandem mass spectra for rA, m^{6}A, m^{6}A$_m$ and their respective stable-isotope-labeled standards, i.e., [$^{13}C_5$]-rA and [D_3]-m^{6}A (b).
