# Supplementary material for: HIV reprograms host m6Am RNA methylome by viral Vpr protein-mediated degradation of PCIF1
Source: Nat Commun. 2021 Sep 20;12:5543. doi: 10.1038/s41467-021-25683-4 (PMC8452764; doi:10.1038/s41467-021-25683-4)
Supplement: Supplementary file 2 — Description of Additional Supplementary Files [file 41467_2021_25683_MOESM2_ESM.pdf]

Description of Additional Supplementary Files

File Name: Supplementary Data 1
Description: shRNA and oligos used in this study.

File Name: Supplementary Data 2
Description: Peak distribution of the m^6Am genes in Jurkat T cells.

File Name: Supplementary Data 3
Description: m^6Am genes with decreased peak density

File Name: Supplementary Data 4
Description: m^6Am genes interacted with HIV under different GO categories
