# Supplementary material for: HIV reprograms host m6Am RNA methylome by viral Vpr protein-mediated degradation of PCIF1
Source: Nat Commun. 2021 Sep 20;12:5543. doi: 10.1038/s41467-021-25683-4 (PMC8452764; doi:10.1038/s41467-021-25683-4)
Supplement: Supplementary file 7 — Reporting Summary [file 41467_2021_25683_MOESM7_ESM.pdf]

nature portfolio

Corresponding author(s): Tariq Rana

Last updated by author(s): Aug 16, 2021

Reporting Summary

Nature Portfolio wishes to improve the reproducibility of the work that we publish. This form provides structure for consistency and transparency in reporting. For further information on Nature Portfolio policies, see our Editorial Policies and the Editorial Policy Checklist.

Statistics

For all statistical analyses, confirm that the following items are present in the figure legend, table legend, main text, or Methods section.

n/a	Confirmed	
☐	☒	The exact sample size (*n*) for each experimental group/condition, given as a discrete number and unit of measurement
☐	☒	A statement on whether measurements were taken from distinct samples or whether the same sample was measured repeatedly
☐	☒	The statistical test(s) used AND whether they are one- or two-sided<br>*Only common tests should be described solely by name; describe more complex techniques in the Methods section.*
☐	☒	A description of all covariates tested
☐	☒	A description of any assumptions or corrections, such as tests of normality and adjustment for multiple comparisons
☐	☒	A full description of the statistical parameters including central tendency (e.g. means) or other basic estimates (e.g. regression coefficient) AND variation (e.g. standard deviation) or associated estimates of uncertainty (e.g. confidence intervals)
☒	☐	For null hypothesis testing, the test statistic (e.g. *F*, *t*, *r*) with confidence intervals, effect sizes, degrees of freedom and *P* value noted<br>*Give P values as exact values whenever suitable.*
☒	☐	For Bayesian analysis, information on the choice of priors and Markov chain Monte Carlo settings
☒	☐	For hierarchical and complex designs, identification of the appropriate level for tests and full reporting of outcomes
☒	☐	Estimates of effect sizes (e.g. Cohen's *d*, Pearson's *r*), indicating how they were calculated

Our web collection on statistics for biologists contains articles on many of the points above.

Software and code

Policy information about availability of computer code

Data collection	no software was used
Data analysis	The comparisons were performed using Graphpad v8.0.1

For manuscripts utilizing custom algorithms or software that are central to the research but not yet described in published literature, software must be made available to editors and reviewers. We strongly encourage code deposition in a community repository (e.g. GitHub). See the Nature Portfolio guidelines for submitting code & software for further information.

Data

Policy information about availability of data

All manuscripts must include a data availability statement. This statement should provide the following information, where applicable:

- Accession codes, unique identifiers, or web links for publicly available datasets
- A description of any restrictions on data availability
- For clinical datasets or third party data, please ensure that the statement adheres to our policy

The accession number for the m6A-MeRIP seq sequencing data reported in this paper is NCBI GEO: GSE154035 (https://www.ncbi.nlm.nih.gov/geo/query/acc.cgi?acc=GSE154035). The accession number for the m6Am-Exo-Seq sequencing data reported in this paper is NCBI GEO: GSE171800 (https://www.ncbi.nlm.nih.gov/geo/query/acc.cgi?acc=GSE171800). The raw data for LC/MS quantification curves of modified RNA and uncropped versions of Western blots are provided in the Source Data file. The coding for m6A-MeRIP and m6Am-Exo-Seq analysis is deposited at Github: https://github.com/KylinKang-bme/m6Am_code, which can also be found at zenodo: https://doi.org/10.5281/zenodo.5173217

Field-specific reporting

Please select the one below that is the best fit for your research. If you are not sure, read the appropriate sections before making your selection.

- [x] Life sciences
- [ ] Behavioural & social sciences
- [ ] Ecological, evolutionary & environmental sciences

For a reference copy of the document with all sections, see nature.com/documents/nr-reporting-summary-flat.pdf

Life sciences study design

All studies must disclose on these points even when the disclosure is negative.

Sample size

All RT-qPCR experiments were repeated three or four times. Three independent biological experiments are sufficient to ensure data reproducibility and P value calculation. P values were calculated with a two-sided unpaired Student's t-test if without specification.

Data exclusions

N/A, no data were excluded.

Replication

Experiments for m6Am-Exo-Seq analysis were performed with two independent biological replicates. The RT-qPCR experiments and ChIP-qPCR experiments in this study were performed with three or four independent biological replicates. We specified the number of replications in the Figure legends. All replication attempts were successful.

Randomization

We plated the cells randomly in multi-well plates, and the cells are randomly collected.

Blinding

All the fastq files were generated independently by the UCSD genomics core without any knowledge of the experimental groups. Because all the data generated in this study were for objective quantitative analyses, the researchers were not blinded for experimental design and outcome assessment.

Reporting for specific materials, systems and methods

We require information from authors about some types of materials, experimental systems and methods used in many studies. Here, indicate whether each material, system or method listed is relevant to your study. If you are not sure if a list item applies to your research, read the appropriate section before selecting a response.

Materials & experimental systems

n/a	Involved in the study	
[ ]	[x]	Antibodies
[ ]	[x]	Eukaryotic cell lines
[x]	[ ]	Palaeontology and archaeology
[x]	[ ]	Animals and other organisms
[x]	[ ]	Human research participants
[x]	[ ]	Clinical data
[x]	[ ]	Dual use research of concern

Methods

n/a	Involved in the study	
[x]	[ ]	ChIP-seq
[x]	[ ]	Flow cytometry
[x]	[ ]	MRI-based neuroimaging

Antibodies

Antibodies used

Antibodies and dilutions were used as follows: anti-PCIF1 antibody at 1:1000 (Proteintech, 16082-1), anti-p24 antibody at 1:1000 (abcam, ab9071), anti-p62 antibody at 1:1000 (abcam, ab56416), anti-Vpr antibody at 1:1000 (Proteintech, 51143-1-AP), anti-Vpu antibody at 1:1000 (NIH AIDS Reagent Program, Cat# 969), anti-Nef antibody at 1:1000 (NIH AIDS Reagent Program, Cat# 1539), anti-Vif antibody at 1:1000 (NIH AIDS Reagent Program, Cat# 6460), anti GAPDH-HRP antibody at 1:5000 (Proteintech, HRP-60004) , mouse anti-FLAG M2 antibody at 1:1000 (Sigma-Aldrich, F1804), anti-HDAC1 antibody at 1:1000 (abcam, ab7028), anti ETS-1 antibody at 1:2000 (Proteintech, 12118-1-AP), anti-GFP-tag antibody at 1:1000 (Proteintech, 50430-2-AP), HA antibody at 1:1000 (Santa-Cruz, sc-805). HRP-conjugated secondary antibodies were used for all the western blots at a dilution of 1:10 (Pierce Fast Western Blotting Blot Kit, 35055).

Validation

All of these antibodies have been validated by ChIP, WB and Co-IP in published research or by the manufacturer. Detailed information can be found at the links or publications listed below:
anti-PCIF1 antibody at 1:1000 (Proteintech, 16082-1): https://www.ptgcn.com/products/PCIF1-Antibody-16082-1-AP.htm
anti-p24 antibody at 1:1000 (abcam, ab9071): https://www.abcam.com/hiv1-p24-antibody-3954a-ab9071.html
anti-p62 antibody at 1:1000 (abcam, ab56416): https://www.abcam.com/sqstm1--p62-antibody-2c11-bsa-and-azide-free-ab56416.html
anti-Vpr antibody at 1:1000 (Proteintech, 51143-1-AP): https://www.ptgcn.com/products/Vpr-Antibody-51143-1-AP.htm
anti-Vpu antibody at 1:1000 (NIH AIDS Reagent Program, Cat# 969): https://www.hivreagentprogram.org/Catalog/HRPPolyclonalAntiserum/ARP-969.aspx

anti-Nef antibody at 1:1000 (NIH AIDS Reagent Program, Cat# 1539): https://www.hivreagentprogram.org/Catalog/HRPMonoclonalAntibodies/ARP-1539.aspx
anti-Vif antibody at 1:1000 (NIH AIDS Reagent Program, Cat# 6460): https://www.hivreagentprogram.org/Catalog/HRPMonoclonalAntibodies/ARP-6460.aspx
anti GAPDH-HRP antibody at 1:5000 (Proteintech, HRP-60004): https://www.ptgcn.com/products/GAPDH-Antibody-HRP-60004.htm
mouse anti-FLAG M2 antibody at 1:1000 (Sigma-Aldrich, F1804): https://www.sigmaaldrich.com/US/en/search/f1804?focus=products&page=1&perPage=30&sort=relevance&term=f1804&type=product_name
anti-HDAC1 antibody at 1:1000 (abcam, ab7028): https://www.abcam.com/hdac1-antibody-ab7028.html
anti ETS-1 antibody at 1:2000 (Proteintech, 12118-1-AP): https://www.ptgcn.com/products/ETS1-Antibody-12118-1-AP.htm
anti-GFP-tag antibody at 1:1000 (Proteintech, 50430-2-AP): https://www.ptgcn.com/products/eGFP-Antibody-50430-2-AP.htm
HA antibody at 1:1000 (Santa-Cruz, sc-805): https://www.scbt.com/zh/p/ha-probe-antibody-y-11
All of these antibodies have been validated by ChIP, WB and Co-IP in published research or by the manufacturer. Detailed information can be found at the links or publications listed below:
anti-PCIF1 antibody at 1:1000 (Proteintech, 16082-1): https://www.ptgcn.com/products/PCIF1-Antibody-16082-1-AP.htm
anti-p24 antibody at 1:1000 (abcam, ab9071): https://www.abcam.com/hiv1-p24-antibody-3954a-ab9071.html
anti-p62 antibody at 1:1000 (abcam, ab56416): https://www.abcam.com/sqstm1--p62-antibody-2c11-bsa-and-azide-free-ab56416.html
anti-Vpr antibody at 1:1000 (Proteintech, 51143-1-AP): https://www.ptgcn.com/products/Vpr-Antibody-51143-1-AP.htm
anti-Vpu antibody at 1:1000 (NIH AIDS Reagent Program, Cat# 969): https://www.hivreagentprogram.org/Catalog/HRPPolyclonalAntiserum/ARP-969.aspx
anti-Nef antibody at 1:1000 (NIH AIDS Reagent Program, Cat# 1539): https://www.hivreagentprogram.org/Catalog/HRPMonoclonalAntibodies/ARP-1539.aspx
anti-Vif antibody at 1:1000 (NIH AIDS Reagent Program, Cat# 6460): https://www.hivreagentprogram.org/Catalog/HRPMonoclonalAntibodies/ARP-6460.aspx
anti GAPDH-HRP antibody at 1:5000 (Proteintech, HRP-60004): https://www.ptgcn.com/products/GAPDH-Antibody-HRP-60004.htm
mouse anti-FLAG M2 antibody at 1:1000 (Sigma-Aldrich, F1804): https://www.sigmaaldrich.com/US/en/search/f1804?focus=products&page=1&perPage=30&sort=relevance&term=f1804&type=product_name
anti-HDAC1 antibody at 1:1000 (abcam, ab7028): https://www.abcam.com/hdac1-antibody-ab7028.html
anti ETS-1 antibody at 1:2000 (Proteintech, 12118-1-AP): https://www.ptgcn.com/products/ETS1-Antibody-12118-1-AP.htm
anti-GFP-tag antibody at 1:1000 (Proteintech, 50430-2-AP): https://www.ptgcn.com/products/eGFP-Antibody-50430-2-AP.htm
HA antibody at 1:1000 (Santa-Cruz, sc-805): https://www.scbt.com/zh/p/ha-probe-antibody-y-11

Eukaryotic cell lines

Policy information about cell lines

Cell line source(s)	Cells are purchased from ATCC. MT4, THP1, and Jurkat cells were cultured in RPMI containing 10% fetal bovine serum (FBS) and 50 uM β-mercaptoethanol (Sigma). HeLa and 293FT cells were cultured in DMEM (Invitrogen) with 10% FBS.
Authentication	All the cell lines are common used cell lines that purchased from ATCC. The cell lines were not recently authenticated.
Mycoplasma contamination	All cell lines used in this study were negative for mycoplasma contamination.
Commonly misidentified lines (See ICLAC register)	No commonly misidentified cell lines were used.
